# *Schistosoma haematobium* infection among school age children in two peri-urban areas of Lusaka, Zambia: A cross-sectional study

**DOI:** 10.1371/journal.pntd.0014013

**Published:** 2026-10-21

**Authors:** Mable M. Mutengo, James Mwansa, Kelly Chisanga, Emmanuel Zulu, Namwiinga Rozaria Mulunda, James Muchinga, Esther Rodríguez, Sergio Sánchez, Lourdes Castro, María J. Perteguer, David Carmena, Javier Sotillo

**Affiliations:** 1 Institute of Basic and Biomedical Sciences, Levy Mwanawasa Medical University, Lusaka, Zambia; 2 Department of Postgraduate and Research, Lusaka Apex Medical University, Lusaka, Zambia; 3 Parasitology Reference and Research Laboratory, Centro Nacional de Microbiologia, Instituto de Salud Carlos III, Majadahonda, Madrid, Spain; 4 CIBERINFEC, Instituto de Salud Carlos III, Madrid, Spain; George Washington University School of Medicine and Health Sciences, UNITED STATES OF AMERICA

## Abstract

**Background:**

Schistosomiasis is one of the most prevalent neglected tropical diseases in sub-Saharan Africa, causing substantial morbidity and millions of disability-adjusted life years (DALYs). Although the WHO aims to eliminate schistosomiasis as a public health problem in several countries by 2030, current epidemiological data on schistosomiasis in Zambia are insufficient to adequately characterize transmission patterns, identify priority intervention areas, and inform the planning and evaluation of control programmes.

**Methods:**

We assessed the prevalence of urogenital schistosomiasis among school-age children in two peri-urban districts of Lusaka (Chongwe and Kafue). A total of 208 participants were enrolled, and infection status was determined using urine filtration microscopy, ELISA, and quantitative PCR (qPCR).

**Results:**

Urogenital schistosomiasis prevalence was significantly higher in Chongwe than in Kafue, as determined by both microscopy and qPCR. By microscopy, 34 of 48 positive cases (70.8%) were from Chongwe and 14 (29.2%) from Kafue (χ² = 4.82; P < 0.05). Similarly, qPCR revealed a prevalence of 73.9% in Chongwe and 27.84% in Kafue (χ² = 14.31; P < 0.01). No significant association was observed between sex and infection status. However, infection was significantly associated with age across the study population.

**Conclusions:**

Our findings demonstrate that urogenital schistosomiasis remains highly prevalent in the peri-urban districts of Chongwe and Kafue in Lusaka Province, Zambia, highlighting ongoing transmission and the need for strengthened surveillance and control efforts. These results provide updated information on the epidemiological situation of urogenital schistosomiasis in Zambia and will support planning and implementation within the WHO NTD agenda.

## Author summary

Urogenital schistosomiasis remains a major public health problem in many parts of sub-Saharan Africa, yet current epidemiological data from Zambia are limited, particularly in peri-urban communities surrounding Lusaka. In this study, we assessed the prevalence of urogenital schistosomiasis among school-age children in two peri-urban districts, Chongwe and Kafue, using complementary parasitological, serological, and molecular diagnostic methods. We found significantly higher infection levels in Chongwe than in Kafue, with quantitative PCR (qPCR) detecting substantially more infections than conventional urine microscopy. Infection was also significantly associated with age but not sex. These findings indicate that urogenital schistosomiasis remains actively transmitted and that reliance on microscopy alone may underestimate the true burden of infection. Our study provides current epidemiological evidence that can inform surveillance, guide targeted control interventions, and support ongoing efforts to eliminate schistosomiasis as a public health problem in Zambia.

## Introduction

Schistosomiasis is a chronic, often debilitating disease, caused by various trematodes of the genus *Schistosoma* [1]. It is estimated that, worldwide, more than 250 million people are infected with *Schistosoma* spp., 90% of whom live in Africa [2]. Furthermore, these parasites cause tens to hundreds of thousands of direct deaths annually [3], while near 800 million people live in high-risk areas and over 250 million people required preventive chemotherapy in 2021 [2,4]. The Global Burden of Disease Study 2016 estimates that schistosomiasis accounts for 1.9 million disability-adjusted life years (DALYs) worldwide [5], although a previous meta-analysis study suggested a severalfold higher burden [6]. Of the six species that can infect humans, *Schistosoma haematobium* causes over half of all infections, leading to urogenital schistosomiasis and various health issues, including urinary and genital disorders [1,2]. Notably, urogenital schistosomiasis has been linked to squamous cell carcinoma of the urinary bladder. Indeed, the WHO International Agency for Research on Cancer (IARC) classifies *S. haematobium* as a Group 1 carcinogen [7,8].

Infection with *S. haematobium* occurs when the cercariae released from the snail intermediate host penetrate the skin of the human host, losing their tail and transforming into schistosomula [1]. After entering the venous blood vessels, the schistosomula are transported to the lungs, then to the heart, reaching the arterial circulation and finally migrating to the venous plexuses around the urinary bladder. Once there, females will produce and shed thousands of eggs daily, which pass through the bladder wall, and are excreted in the urine. However, a proportion of the eggs become trapped within the bladder, ureters, and genital tract where they induce granulomatous inflammation and other host immune responses that are responsible for much of the disease pathology. Eggs that reach freshwater hatch into miracidia, which subsequently infect snails of the genus *Bulinus*, the intermediate hosts of *S. haematobium*.

Although both intestinal and urinary schistosomiasis are endemic in all 10 provinces of Zambia, the most prevalent species is *S. haematobium* [9]. Higher prevalences are usually found in rural districts near rivers and lakes [10], although schistosomiasis is also prevalent in many peri-urban areas due to poor water supply infrastructure [11]. For instance, the overall prevalence of schistosomiasis in children between ages of 5 and 17 years in a peri-urban area outside Lusaka, reported in the early 2010s, was 20.7% [11], whereas studies conducted in the 2000s in other areas from the Siavonga and Lusaka districts reported prevalence rates ranging from 8% to 12% [12]. However, most epidemiological studies have employed microscopy as the reference diagnostic method, which has reduced sensitivity, particularly for detecting low-intensity infections, and may therefore underestimate the true burden of disease [9,12]. A recently published meta-analysis showed that prevalence of schistosomiasis by *S. haematobium* declined between 1990–2010, most likely due to diagnostic and treatment efforts from the Zambian Bilharzia Control Programme (ZBCP), but has significantly increased in the last decade, which was attributed to a lack of monitoring and implementation of surveillance programs [9,13]. Despite several epidemiological surveys conducted during the early 2000s, current epidemiological data on schistosomiasis in Zambia remain limited, restricting evidence-based disease mapping, identification of priority intervention areas, and evaluation of schistosomiasis control programmes. This has led to inefficient coverage of Mass Drug Administration (MDA) interventions and increase of other diseases such as HIV, which has been associated to schistosome infection in urban and peri-urban areas of Lusaka [14].

In the present study, we conducted an epidemiological survey in two different peri-urban areas of Lusaka Province: Chongwe and Kafue, selected on the basis of historical mapping studies that identified them as high-transmission areas (>50% and near 90% prevalence in Chongwe and Kafue, respectively) [15]. Given the limited sensitivity of urine microscopy, particularly in low-intensity infections, quantitative PCR (qPCR) was incorporated to improve the detection of *S. haematobium*. A serological assay was also employed to assess previous exposure to *Schistosoma* spp. and provide complementary information on population-level exposure. For this purpose, an in-house ELISA using a recombinant *S. haematobium* antigen (*Sh*-TSP-2)was employed rather than the more traditional soluble egg antigen (SEA), since SEA is a crude, poorly standardised antigen preparation that is well documented to cross-react with other helminth infections and is costly and difficult to produce consistently across laboratories, whereas defined recombinant antigens allow for a reproducible reagent with improved specificity [16]. This study aimed to determine the prevalence of urogenital schistosomiasis and characterize infection patterns among school-age children in two peri-urban districts of Lusaka Province, addressing an important gap in the current epidemiological evidence on schistosomiasis in Zambia.

## Methods

### Ethics statement

The research was approved by the Levy Mwanawasa Medical University Research Ethics Committee (LMMU-REC 000054/23) and the authority to conduct research was obtained from the National Health Research Authority (NHRA0001/23/04/2023). Prior to commencement of the study, permission was also sought from the Lusaka Province Health office and the Ministry of Education. Written informed consent was obtained from the parents or legal guardians of all participating children, and assent was obtained from the children themselves when appropriate. Participation was entirely voluntary, and care was taken to ensure that children and their guardians understood that they could withdraw from the study at any time without any consequence. The confidentiality and privacy of all participants were rigorously protected. All infected participants were treated with praziquantel (40mg/kg body weight) according to national treatment guidelines.

### Study area and population

The study was conducted in two peri-urban regions of Lusaka Province namely Chongwe and Kafue Districts (Fig 1). Chongwe District is located 40 kilometres east of Lusaka city, the capital city of Zambia. The district has a population of 313,389 inhabitants with most of its residents engaged in farming and livestock rearing. Chongwe District is traversed by the Chongwe River, which is a key water source for both domestic use and agriculture for most households. However, the river and its tributaries also pose a risk for waterborne diseases such as schistosomiasis.

**Fig 1:**
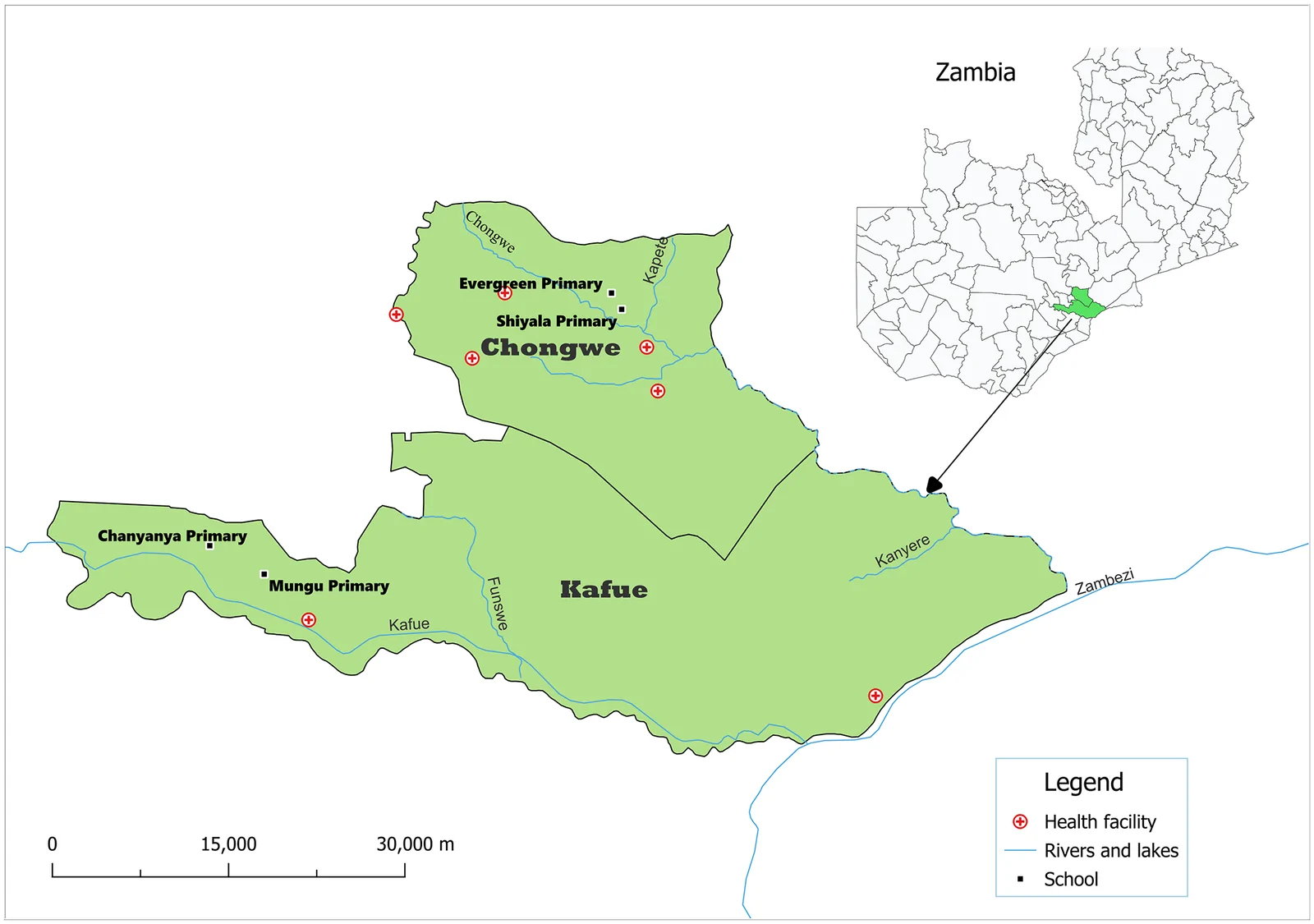
Map of the Chongwe and Kafue Districts in Lusaka Province showing the location of the sampled primary schools. The base layer of the map was obtained from https://gadm.org/maps.html. The GADM data is freely available for academic and non-commercial use and is compatible with the CC BY 4.0 license.

Kafue District is situated in the Southern part of Lusaka Province at the tip of Lusaka District and is located 45 kilometres away from the capital city, Lusaka. It has a population of 220,000 inhabitants. The district shares borders with Chongwe District to the northeast and Lusaka (urban) to the North, while its southwestern part borders Mazabuka and Chirundu districts. Kafue district has two major rivers, the Kafue and the Zambezi River running through the district with a sizable number of perennial rivers. This abundance of water creates several pockets of recreational water bodies where children go to swim and can become infected with bilharzia. Most of the district population is settled along the Zambezi and Kafue River where agriculture and fishing are important livelihood activities.

The minimum sample size was determined using the single population proportion formula. An expected prevalence of 10% was assumed based on a previous study of *S. haematobium* among children in Lusaka and Siavonga Districts, Zambia [12]. A 95% confidence level and a margin of error of 5% were used, yielding an initial sample size of 139 participants. Because participants were recruited from two study sites, a design effect of 1.5 was applied to account for potential clustering within sites, resulting in a target sample size of 208 participants. Participants were school-age children who were selected using a systematic random sampling method. They were recruited from two schools in Chongwe namely Evergreen and Shiyala basic schools and Kafue District namely Chanyanya and Mungu Basic Schools (Fig 1). The GPS coordinates of the four participating schools were as follows: Evergreen Primary School (15.2672°S, 28.64602°E) and Shiyala Primary School (15.2921°S, 28.66161°E) in Chongwe District; Chanyanya Primary School (15.6535°S, 28.03168°E) and Mungu Primary School (15.6971°S, 28.11493°E) in Kafue District. On the day of sampling, all eligible school-age children present at each participating school were assembled. The sampling interval (k) was calculated as the ratio of the number of eligible children present (N) to the required sample size (n). Following a random starting point, every kth eligible child was selected until the required sample size was attained [17].

### Parasitological and biochemical analysis

A single urine sample was collected from each of the recruited participants and analysed for visible haematuria, microscopic leukocytes, biochemistry protein parameters and presence of *S. haematobium* eggs. Visible haematuria was assessed macroscopically, while microhaematuria, urinary leukocyte esterase, and urinary protein were assessed using Multistix 10 SG reagent strips (Bayer, Germany) according to the manufacturer’s instructions. Results were recorded semi-quantitatively as negative, trace, 1+, 2+, or 3+. For microscopy detection of *S. haematobium*, the urine filtration method was used. Briefly, each urine sample was vigorously mixed and 10 ml was drawn into a syringe and passed through a nucleopore membrane filter, with a pore size of 20 μm [18]. The filter was then placed onto a microscope slide and examined under 10x and 40×objective lenses for the presence of *S. haematobium* eggs. The eggs were counted manually, and infection load was recorded as the number of eggs per 10 ml of urine.

#### ELISA.

The recombinant protein *Sh*-TSP-2 *was* expressed and purified as described previously [16]. In brief, *E. coli* BL21(DE3) harbouring the protein-encoding plasmid was cultured overnight at 37°C with agitation at 200 rpm in 10 ml of Luria broth supplemented with 50 μg/ml kanamycin (LB_kan_). Subsequently, the overnight culture was inoculated (1/100) into 500 ml of fresh LB_kan_ and grown at 37°C with shaking until reaching an OD_600_ of 0.5–1.0. Protein expression was induced by adding 1 mM isopropyl beta-D-1-thiogalactopyranoside (IPTG) and allowing the culture to incubate for an additional 24 h. After induction, cells were harvested by centrifugation at 8,000 *g* for 20 minutes at 4°C, then resuspended in 50 ml of lysis buffer (50 mM sodium phosphate pH 8, 40 mM imidazole, 300 mM NaCl), and subjected to three cycles of freeze/thawing followed by sonication at 4°C. The bacterial lysate was then centrifuged at 20,000 *g* for 20 min at 4°C, and the resulting supernatant was collected and stored at –80°C.

Recombinant proteins were purified using an ÄKTA Pure UPC FPLC system (GE Healthcare, IL, USA) equipped with Ni2+ immobilized metal ion affinity chromatography (IMAC) at 4°C. For this, the recombinant protein solutions were diluted 1:4 in lysis buffer, filtered through a 0.45 μm membrane, and loaded onto a 1 ml His-Trap IMAC column (GE Healthcare) pre-equilibrated with lysis buffer at a flow rate of 1 ml/min. Bound proteins were washed with 10 column volumes of lysis buffer, followed by elution with a linear gradient of imidazole (100–500 mM) in lysis buffer. Fractions containing the highest purity of the target protein were pooled and subjected to buffer exchange into PBS using an Amicon Ultra-15 centrifugal filter with a 3 kDa molecular weight cut-off. The identity of the purified proteins was confirmed through SDS-PAGE and Western blot analysis utilizing anti-His monoclonal antibodies (Invitrogen, CA, USA).

Nunc MicroWell 96-Well Medisorp Microplates (Thermo Fisher Scientific, MA, USA) were coated overnight at 4°C with a solution containing 2 μg/ml of *Sh*-TSP-2 in 0.1 M Na_2_CO_3_/NaHCO_3_ buffer at pH 9.6. The following day, the plates were washed with PBS-Tween-20 at 0.05% (PBST) and blocked for 2 h at room temperature with 100 μl of PBST/5% skimmed milk powder. Following blocking, 50 μl of urine (diluted 1:10 in PBST) were added to the respective wells and allowed to incubate overnight at 4°C. The following day, the plates were washed with PBST and subsequently incubated for 1 h at room temperature with 100 μl of goat anti-human IgG-HRP (Sigma, MA, USA) diluted to 1:5,000 in PBST. After another round of washing with PBST, the plates were developed using 3,3′,5,5′-Tetramethylbenzidine (TMB), and the reaction was halted with 0.5 M sulfuric acid. The absorbance was measured at a wavelength of 492 nm using a Multiskan FC (Thermo Fisher Scientific) microplate reader.

### DNA extraction and real-time PCR

DNA was extracted using the Quick-DNA Urine Kit (Zymo Research, CA, USA) following the manufacturer’s instructions. Briefly, 280 μl of Urine Conditioning Buffer was added to 4 ml of urine, followed by the addition of 10 μl of Clearing Beads. The samples were, then, mixed and centrifuged at 3,000 *g* for 15 min. Subsequently, the Tough-to-Lyse Samples protocol was followed. The urine supernatant was decanted, leaving behind 200 μl of pellet. The pellet was transferred to a ZR BashingBead Lysis Tube (0.1 & 0.5 mm), and 600 μl of BashingBead Buffer was added. The mixture was vortexed in a Vortex-Genie 2 mixer for 5 min at maximum speed. Tubes were then centrifuged at 10,000 *g* for 1 min, and 400 μl of the sample was applied to a Zymo-Spin III-F Filter and centrifuged again at 10,000 *g* for 1 min. Following this, 1,200 μl of Genomic Lysis Buffer was added to the flow-through, and the mixture was centrifuged through a Zymo-Spin IC-S Column. The column was washed successively with 200 μl of Urine DNA Prep Buffer, and 700 μL and 200 μl of Urine DNA Wash Buffer. The columns were then transferred to clean 1.5 ml microcentrifuge tubes, and DNA was eluted by adding 50 µl of deionized molecular biology-grade water directly to the column matrix, followed by centrifugation at 14,000 *g* for 1 min. DNA concentration and purity were estimated using a Nanodrop One spectrophotometer (Thermo Fisher Scientific) as a routine quality‑control measure following DNA extraction.

Reactions targeting the Dra1 tandem repeat [19] were performed in duplicate as follows: 20 µl reactions contained 5 μl of DNA, 10 μl of Quantimix easy kit (Biotools, Madrid, Spain), 1 ul of PrimeTime standard qPCR Assay that included 500 nM of primers Fwd (5´–GATCTCACCTATCAGACGAA–3´) and Rv (5´–TCACAACGATACGACCAAC–3´) each and 300 nM of probe (FAM- TGTTGGTGGAAGTGCCTGTTTCGCAA-IowaBlack Fluorescent Quencher). To monitor for PCR inhibition and reduce the risk of false-negative results, an internal amplification control (IAC) targeting the human 18S rRNA gene was multiplexed into each reaction. 18S *H. sapiens* assay included 500 nM of primers Fwd (5´–GACGGTATCTGATCGTCTTC–3´) and Rv (5´–GAGCCGCCTGGATACCGC-3´) each and 300 nM of probe (HEX-TCGCTCTGGTCCGTCTTG -IowaBlack Fluorescent Quencher). This 18S internal amplification control (IAC) is used routinely in our laboratory at the Instituto de Salud Carlos III (ISCIII) for both urine and blood/serum samples as part of our standard diagnostic and research workflow; its performance is therefore well established through routine laboratory use rather than through study-specific titration or optimisation experiments. Samples were considered positive for *S. haematobium* when the Dra1 target amplified with a cycle threshold (Ct) value ≤ 40; as a quality-control measure, samples yielding Ct values above 35 were subjected to more detailed scrutiny and were repeated to confirm the amplification signal before being reported as positive; successful amplification of the 18S IAC in each reaction confirmed the absence of significant PCR inhibition. Extraction blanks and qPCR blanks were used as control for cut-off calculation. A Thermocycler (Rotor Gene Q, Qiagen, Germany) setting consisted of an initial step of 2 min at 95°C followed by 45 cycles of 15 s at 95°C and 60 s at 60°C.

### Statistical analysis

The prevalence of infection and its 95% confidence interval (CI) were calculated using an in-house Python script, incorporating the Z-value for a 95% confidence level (https://github.com/JavierSotillo/Scripts/blob/main/Prevalence_CI_Calculator.py). All other statistical tests were performed using GraphPad Prism v 10.2. Associations between infection status and categorical variables (district, sex, and age group) were assessed using the Chi-square test. To calculate the correlation between age and the number of *Schistosoma* eggs in infected children, a Spearman correlation test was performed. To compare the mean age of male and female participants, as well as the median number of eggs in each location, a two-tailed Mann-Whitney test was employed. *P*-values < 0.05 were considered as significant.

Ct values were not systematically recorded as a continuous variable for all samples; qPCR results were therefore treated as a binary outcome (positive/negative) based on the pre-specified Ct ≤ 40 cut-off described above, and intensity-based comparisons between qPCR and egg counts were not performed. Because no single test can serve as a true gold standard for *S. haematobium* infection given the imperfect sensitivity of microscopy and the imperfect specificity of ELISA and qPCR, diagnostic performance was additionally assessed against a composite reference standard (CRS) as described previously [20,21]. Participants were classified as infected by the CRS if they had a positive microscopy result, or, among microscopy-negative participants, if at least two of the remaining three index tests (microhaematuria, ELISA, qPCR) were concordantly positive. Sensitivity, specificity, positive predictive value (PPV) and negative predictive value (NPV) were calculated for each of the four index tests against the CRS, with 95% CI calculated using the normal (Wald) approximation.

## Results

### Demographic and biochemical characteristics of the study participants

A total of 208 participants from Chongwe and Kafue districts were enrolled into the study (111 from Chongwe and 97 from Kafue) (S1 Table). From these, a total of 99 were females and 109 were males, and their mean age was 10.29 ± 2.95 and 10.83 ± 2.79, respectively (Table 1). No significant difference between the males and females in terms of age was found (*P* = 0.184). From the 111 participants enrolled in the Chongwe district, a total of 55 were females and 56 were males, and their mean age was 11.58 ± 2.99 and 11.96 ± 2.77, respectively (Table 1). Furthermore, from the 97 participants enrolled in the Kafue district, a total of 44 were females and 53 were males, and their mean age was 8.68 ± 1.96 and 9.62 ± 2.27, respectively (Table 1). Mean age differences were statistically significant in the case of Kafue district (*P* < 0.05).

**Table 1:** Number of male and female and mean age of children enrolled in the study. Asterisks (*) denote significancy (*P* < 0.05).

District	Females (% of the total)	Males (% of the total)	Female Mean Age (mean ± SD)	Male Mean Age (mean ± SD)
**Chongwe**	55 (49.55%)	56 (50.45%)	11.58 ± 2.99	11.96 ± 2.77
**Kafue**	44 (45.36%)	53 (54.64%)	8.68 ± 1.96 *	9.62 ± 2.27 *
**TOTAL**	99 (47.60%)	109 (52.40%)	10.29 ± 2.95	10.83 ± 2.79

### Urinary biochemical findings

Visible haematuria was observed in only 22 (10.6%) from the total participants, whereas 53 participants (25.5%) were positive for microhaematuria by Multistix 10 SG. From these, 3 of them showed traces, and 16, 13 and 21 were categorized as 1 + , 2+ and 3 + based on the band intensities (S1 Table).

Leukocyte positivity was included as a non‑specific indicator of urinary tract inflammation to contextualize haematuria findings and was not interpreted as a marker of *S. haematobium* infection per se. Leukocytes were detected in the urine of 40 participants (19.23%), with intensities varying from trace (7 participants), 1+ (26 participants), 2+ (6 participants) and 3+ (1 participant). Only 29 of these leukocyte positive participants also showed microhaematuria (S1 Table).

### District-level variation in *S. haematobium* infection across diagnostic methods

The prevalence of *S. haematobium* infection differed between districts across all diagnostic methods used (Table 2). By microscopy, infection was detected in 48 of 208 children, corresponding to an overall prevalence of 23.1% (95% CI: 17.3–28.8). A higher proportion of infections was observed in Chongwe district (34/48, 70.8%) compared to Kafue district (14/48, 29.2%), with a statistically significant association between infection status and district location (χ^2^ = 4.82; P < 0.05). qPCR identified 109 positive cases, corresponding to a prevalence of 52.4% (95% CI: 45.6–59.2). Prevalence was significantly higher in Chongwe district (73.9%, 95% CI: 65.7–82.1) than in Kafue district (27.84%, 95% CI: 18.92–36.75) (χ^2^ = 14.31; P < 0.01).

**Table 2:** Prevalence of *Schistosoma haematobium* infection in Chongwe and Kafue districts determined by microscopy. qPCR. and serology (ELISA). with associations to geographic location. Asterisks (*) denote significancy (*P* < 0.05).

District	No. examined (%)	Microscopy		qPCR		ELISA	
		No. positive	% Prevalence (95% CI)	No. positive	% Prevalence (95% CI)	No. positive	% Prevalence (95% CI)
**Chongwe**	111 (53.36)	34	30.63 (22.06-39.21)	82	73.87 (65.70-82.05)	51	45.95 (36.67-55.22)
**Kafue**	97 (46.34)	14	14.43 (7.44-21.43)	27	27.84 (18.92-36.75)	49	50.52 (40.57-60.47)
**Difference (χ** ^**2**^ **. *P*-value)**	–	–	χ^2^ =4.82 *P* < 0.05 *	–	χ2 =14.31 P < 0.01 *	–	χ^2^ =0.15 *P* =0.69
**Overall**	**208 (100)**	**48**	**23.08 (17.35-28.80)**	**109**	**52.40 (45.62-59.19)**	**100**	**48.08 (41.29-54.87)**

In contrast, ELISA detected 100 positive cases, corresponding to an overall prevalence of 48.1% (95% CI: 41.3–54.9). Prevalence did not differ significantly between Chongwe (45.9%, 95% CI: 36.7–55.2) and Kafue district (50.5%, 95% CI: 40.6–60.5) (χ^2^ = 0.15; P = 0.69).

Among microscopy-positive participants, the median egg count was 54.5 eggs per 10 ml of urine (95% CI: 45.0–79.0), with no significant difference between districts. No significant correlation was observed between age and egg count (Spearman’s ρ = -0.016, P = 0.904).

### District-level differences in the influence of sex and age on *Schistosoma* infection

When analysing microscopy results by sex, out of the 48 schistosomiasis positive children, 31 (14.9%; 95% CI = 10.1–19.7) were males and 17 (8.2%; 95% CI = 4.5–11.9) were females. As expected, higher prevalences were observed among males (29.8%; 95% CI = 23.6–36.0) than females (22.6%; 95% CI = 16.9–28.3) by qPCR and ELISA (26.0%; 95% CI = 20.0–31.9 and 22.1%; 95% CI = 16.5–27.7). However, no significant association between *Schistosoma* positivity and sex was observed (Table 3).

**Table 3:** Prevalence of *Schistosoma haematobium* infection in Chongwe and Kafue districts determined by microscopy, qPCR, and serology (ELISA). with associations to sex.

Sex	No. examined (%)	Microscopy		qPCR		ELISA	
		No. positive	% Prevalence (95% CI)	No. positive	% Prevalence (95% CI)	No. positive	% Prevalence (95% CI)
**Overall**	**208 (100)**	**48**	**23.08 (17.35-28.80)**	**109**	**52.40 (45.62-59.19)**	**100**	**48.08 (41.29-54.87)**
Female	99 (47.60)	17	8.17 (4.45-11.90)	47	22.60 (16.91-28.28)	46	22.12 (16.48-27.66)
Male	109 (52.40)	31	14.90 (10.06-19.74)	62	29.81 (23.59-36.02)	54	25.96 (20-31.92)
Difference (χ^2^. *P*-value)	–	–	χ^2^ = 2.33. *P* = 0.12	–	χ^2^ = 0.57. *P* = 0.44	–	χ^2^ =0.06. *P* =0.79
**Chongwe**	**111 (100)**	**34**	**30.63 (22.06-39.21)**	**82**	**73.87 (65.70-82.05)**	**51**	**45.95 (36.67-55.22)**
Female	55 (49.54)	14	12.61 (6.44-18.79)	38	34.23 (25.41-43.06)	25	22.52 (14.75-30.29)
Male	56 (50.45)	20	18.02 (10.87-25.17)	44	39.64 (30.54-48.74)	26	23.42 (15.54-31.30)
Difference (χ^2^. *P*-value)	–	–	χ^2^ = 0.73. *P* = 0.39	–	χ^2^ = 0.19. *P* = 0.66	–	χ^2^ =0.003. *P* =0.95
**Kafue**	**97 (100)**	**14**	**14.43 (7.44-21.43)**	**27**	**27.84 (18.92-36.75)**	**49**	**50.52 (40.57-60.47)**
Female	44 (45.36)	3	3.09 (-0.35-6.54)	9	9.28 (3.50-15.05)	21	21.65 (13.45-29.85)
Male	53 (54.64)	11	11.34 (5.03-17.65)	18	18.56 (10.82-26.29)	28	28.87 (19.85-37.88)
Difference (χ^2^. *P*-value)	–	–	χ^2^ = 2.82. *P* = 0.09	–	χ^2^ = 1.2. *P* = 0.26	–	χ^2^ =0.08. *P* = 0.77

Similar results were observed when analysing microscopy results by sex and location. Among the 111 participants in Chongwe, 34 were positive for *S. haematobium* eggs (20 males and 14 females) (Table 3). Interestingly, among the 97 participants from Kafue, 14 were positive by microscopy (11 males and only 3 females). However, no statistically significant difference in the proportion of *S. haematobium* egg-positive cases between males and females was observed in either Chongwe or Kafue (Table 3). Based on the microscopy results, the overall median egg count among all positive children was 54.5 (95% CI: 45.0–79.0) corresponding to a median of 56 (95% CI: 36–107) for males and of 49 (95% CI: 43.0 to 103.0) for females. No significant difference between the median number of eggs between males and females was observed (*P* = 0.7448).

Similarly, among the 82 qPCR-positive children from Chongwe (*n* = 111), 44 were males and 38 females, while in Kafue, 18 males (66.7%) and 9 (33.3%) females were positive out of 97 participants (Table 3). In the case of ELISA, similar prevalences were observed for males and females in both districts (22.5–23.4% and 21.7–28.9%, respectively). No significant association between *Schistosoma* infection and sex was observed within individual districts when prevalence was analysed by qPCR or ELISA.

A statistically significant association between age and *S. haematobium* infection (upon qPCR results) was identified across both study locations (*P* < 0.0001). The mean age of infected children was 11.52 ± 2.97 years, compared to 9.81 ± 2.48 years among uninfected children. This trend was also evident in Kafue (p < 0.05), where the mean age of infected children was 10.32 ± 2.86 years, versus 8.95 ± 1.89 years for those uninfected. However, no significant age-related difference was observed in Chongwe.

### Diagnostic performance against a composite reference standard

To further evaluate the performance of each diagnostic test, participants were classified as infected using a composite reference standard (CRS) combining microscopy, microhaematuria, ELISA and qPCR. Using this approach, 80 of 208 participants (38.5%) were classified as infected. Against this CRS, qPCR showed the highest sensitivity (98.8%, 95% CI: 96.3–100.0) but comparatively lower specificity (76.6%, 95% CI: 69.2–83.9), while microscopy showed the highest specificity and PPV (both 100%) but the lowest sensitivity among the four tests (60.0%, 95% CI: 49.3–70.7). ELISA showed intermediate sensitivity (73.8%, 95% CI: 64.1–83.4) and specificity (68.0%, 95% CI: 59.9–76.1), while microhaematuria showed high specificity (98.4%, 95% CI: 96.3–100.0) but moderate sensitivity (63.7%, 95% CI: 53.2–74.3) (Table 4).

**Table 4:** Diagnostic performance of microscopy, qPCR, ELISA and Multistix 10 SG (microhaematuria) for the detection of *Schistosoma haematobium* infection, using a composite reference standard.

Method	% Sensitivity (95% CI)	% Specificity (95% CI)	% PPV (95% CI)	% NPV (95% CI)
**Microscopy**	60.0 (49.3–70.7)	100.0 (–)	100.0 (–)	80.0 (73.8–86.2)
**qPCR**	98.8 (96.3–100.0)	76.6 (69.2–83.9)	72.5 (64.1–80.9)	99.0 (97.0–100.0)
**ELISA**	73.8 (64.1–83.4)	68.0 (59.9–76.1)	59.0 (49.4–68.6)	80.6 (73.1–88.0)
**Haemastix (microhaematuria)**	63.7 (53.2–74.3)	98.4 (96.3–100.0)	96.2 (91.1–100.0)	81.3 (75.2–87.4)

## Discussion

Urogenital schistosomiasis continues to pose a substantial public health challenge in Zambia, and reliable, up-to-date prevalence data are essential to guide the strategic deployment of MDA programmes and to track progress toward the WHO’s 2030 targets for NTD elimination [22]. To contribute to this effort, we assessed the prevalence of urogenital schistosomiasis in two peri-urban districts of Lusaka Province (Chongwe and Kafue) using different diagnostic approaches. Our findings show that, overall, the qPCR approach identified a higher prevalence (52.4%; 95% CI: 45.6–59.2) than conventional microscopy (23.1%; 95% CI: 17.3–28.8). This aligns with previous studies, as qPCR is widely recognized for its superior diagnostic performance over microscopy, particularly due to its ability to detect low-intensity infections and trace amounts of parasite DNA that may be missed by traditional parasitological methods [23,24]. Here, trace amounts of parasite DNA refer to cell-free or fragmented *Schistosoma* genomic DNA that may be present in urine independently of visible, intact eggs, for example released from adult worms residing in the perivesical venous plexus, or from eggs degrading within the bladder or ureteral wall without being fully excreted; because qPCR amplifies short target sequences rather than requiring a morphologically identifiable egg, it can detect infection even when egg output is too low or irregular to be captured by urine filtration microscopy. In contrast, ELISA yielded a slightly lower prevalence relative to qPCR (48.1%; 95% CI: 41.3–54.9), consistent with its role as a marker of past or cumulative exposure rather than a direct measure of current infection intensity. Furthermore, ELISA seroprevalence exceeded the prevalence indicated by microscopy across both districts, which is expected given that this assay detects antibodies generated in response to past or current exposure to *Schistosoma* spp., rather than active infection per se. Anti-schistosome antibodies can persist in serum for several months, and in some cases longer, after successful treatment, limiting the ability of ELISA alone to distinguish between past exposure and current active infection [25–27]. This distinction is particularly relevant in Kafue district, where ELISA seroprevalence was proportionally high relative to the comparatively low infection rates detected by microscopy and qPCR. According to the National NTD programme at the Zambian Ministry of Health, the last countrywide MDA campaign, which included both Chongwe and Kafue districts, was conducted in 2020. Residual antibody responses from this campaign, together with any subsequent, non-MDA anthelminthic treatment (e.g., for other helminth infections) or ongoing low-level transmission, may therefore contribute to the relatively high ELISA seroprevalence observed in both districts (including in Kafue, where current parasitological and molecular positivity was comparatively low). Accordingly, ELISA results in this study should be interpreted as an indicator of cumulative population-level exposure to *Schistosoma* spp. rather than a direct measure of current infection status, complementing rather than duplicating the parasitological and molecular findings. By “cumulative” exposure, we mean that a positive ELISA result reflects the total history of antigenic stimulation from *Schistosoma* spp. antigens, rather than the presence of a currently patent infection: because IgG antibodies against *Sh*-TSP-2 can persist for many months to years after the parasite itself is no longer present, ELISA cannot distinguish whether a positive signal arises from a recently acquired infection or one cleared long ago; both contribute to the same measurable antibody response.

Because reliance on a single reference test can bias estimates of diagnostic accuracy, particularly given the known limitations of microscopy for detecting low-intensity infections and of ELISA for distinguishing past from current exposure, we additionally evaluated test performance against a CRS integrating microscopy, microhaematuria, ELISA and qPCR. Consistent with its high analytical sensitivity for detecting parasite DNA, qPCR showed the highest sensitivity against the CRS (98.8%), in line with previous studies using the same Dra1-based assay, which similarly reported qPCR detecting substantially more infections than microscopy while showing comparatively lower specificity against combined reference standards [24]. This sensitivity–specificity trade-off is consistent with recent large-scale diagnostic accuracy studies concluding that no single test performs optimally across all settings, and that combining diagnostic approaches improves overall accuracy [28]. qPCR’s comparatively lower specificity (76.6%) suggests the assay may also detect residual or non-viable parasite DNA in individuals not currently harbouring an active infection, consistent with reports of prolonged real-time PCR positivity in urine for many months after successful praziquantel treatment [29]. Although samples with Ct values above 35 were subjected to repeat testing to confirm the amplification signal (see Methods), we cannot fully rule out that some qPCR-positive results in this higher Ct range reflect trace or non-specific amplification rather than robust evidence of active infection, in the absence of a standard curve for quantitative interpretation. These findings are consistent with the expected hierarchy of analytical sensitivity among the three diagnostic approaches used in this study, namely microscopy < ELISA < qPCR: microscopy requires the presence of intact, excreted eggs; ELISA detects an antibody response that develops only after sufficient antigenic stimulus; whereas qPCR can detect trace amounts of parasite DNA independently of egg excretion, giving it the highest analytical sensitivity of the three methods. Microscopy retained the highest specificity and PPV, consistent with its established role as a highly specific, if insensitive, parasitological method. Because each test contributes to the CRS itself, these estimates are subject to incorporation bias, most evidently for microscopy, whose specificity and PPV are inflated toward 100% by construction, and should be interpreted as indicative rather than definitive measures of absolute diagnostic accuracy. Incorporation bias may also arise from the inclusion of ELISA among the four index tests contributing to the CRS: because ELISA cannot distinguish between past and current infection, its concordance with one other positive index test could classify some participants with only past exposure as CRS-positive, which may inflate the apparent sensitivity, and/or deflate the apparent specificity, of the remaining tests (particularly qPCR and microhaematuria) to an extent that cannot be precisely quantified with the present data. Nonetheless, these findings reinforce the complementary value of combining parasitological, molecular and serological approaches to accurately characterise *S. haematobium* infection status in this population.

Interestingly, a significant association was observed between age and infection status among the studied children. Overall, the mean age of infected children was 11.52 ± 2.97 years, compared to 9.81 ± 2.48 years among uninfected children. In Kafue specifically, mean ages were 10.32 ± 2.86 years and 8.95 ± 1.89 years for infected and uninfected participants, respectively. The influence of age on *Schistosoma* infection has been well documented [30–32]. For example, Lamberti *et al*. reported that *Schistosoma* spp. infections peaked at ages 11–12 in Uganda [32], while a meta-analysis on *S. mansoni* risk factors identified the 13–16 age group as highest risk, followed by 10–12 years [31]. Additionally, older participants may have developed partial acquired immunity, which influences infection dynamics and shapes the age–intensity curves observed for *S. haematobium* [33]. In addition to antibody persistence, the role of treatment-induced immune responses should also be considered when interpreting these findings. While Praziquantel is effective in eliminating adult worms, parasite death results in the release of schistosome antigens that can stimulate host immune responses and contribute to the development of partial protective immunity against reinfection [34]. Consequently, infection patterns observed in endemic communities likely reflect a combination of cumulative water exposure, naturally acquired immunity, and previous treatment history.

Both microscopy and qPCR analyses also revealed a statistically significant association between geographic location and the prevalence of urogenital schistosomiasis. Prevalence rates were markedly higher in Chongwe compared to Kafue (30.6% vs. 14.4% by microscopy; 73.9% vs. 27.8% by qPCR), with differences reaching statistical significance (*P* < 0.05). These findings suggest that the observed disparity in infection rates between the two districts is unlikely due to chance and instead reflects distinct schistosomiasis risk profiles. In Zambia, schistosomiasis is typically more prevalent in rural areas located near water bodies such as rivers and lakes [10]. However, peri-urban regions are also affected, largely due to inadequate water infrastructure [11]. For instance, in a peri-urban community near Lusaka, the infection rate among children aged 5–17 years was reported at 20.7% [11], while other locations within the Siavonga and Lusaka districts showed prevalence rates ranging from 8% to 12% [12]. In both Chongwe and Kafue, transmission among school-age children is likely driven primarily by recreational water contact, such as swimming and playing in infested water bodies, and to a lesser extent by domestic activities including bathing, washing, and collecting water. Interestingly, previous studies conducted in these same districts also reported a higher prevalence of the zoonotic protozoan *Cryptosporidium parvum* among schoolchildren in Chongwe than in Kafue [35], suggesting that environmental and behavioural factors may similarly influence the transmission of multiple waterborne parasitic diseases [35].

We acknowledge that the current literature on *Schistosoma* hybridisation requires careful, balanced interpretation. We define hybrids here as offspring resulting from interbreeding between *S. haematobium* and closely related species within the *S. haematobium* group, which can arise both through recent, ongoing hybridisation events and through ancient introgression whose genomic signatures persist in present-day populations without necessarily reflecting active, contemporary zoonotic transmission [36]. Indeed, large-scale genomic analyses of West African *S. haematobium* populations have found evidence of ancient introgression from *S. bovis* but no clear signal of ongoing hybridisation, cautioning against interpreting the presence of hybrid genetic markers alone as evidence of an active zoonotic reservoir. For Zambia specifically, *S. bovis* and *S. curassoni* are not known to occur, and the livestock-associated species of greatest relevance in this region is *S. mattheei*, which has been confirmed to hybridise with *S. haematobium* in neighbouring Malawi, with limited but growing evidence of zoonotic spill-over from cattle [37]. However, very few animals in the region have been confirmed infected with *S. haematobium* or *S. haematobium*-*mattheei* hybrids, and substantial uncertainty remains regarding the extent, direction, and epidemiological significance of zoonotic transmission in this part of southern Africa. These considerations underscore the importance of incorporating a One Health perspective, including molecular surveillance of livestock reservoirs and intermediate snail hosts, into future schistosomiasis control and elimination strategies in Zambia.

This study presents several limitations that should be taken into account when interpreting the findings and drawing conclusions. Firstly, the survey was restricted to school-age children in only two districts of Lusaka Province, which may limit the generalizability of the prevalence to the broader Zambian context. Second, the cross-sectional study design did not permit assessment of temporal changes in infection prevalence, seasonal variation in transmission, or reinfection patterns. Finally, although microscopy, ELISA, and qPCR were used as complementary diagnostic approaches, each method has inherent strengths and limitations. Consequently, differences in diagnostic performance should be considered when comparing prevalence estimates across methods. Additionally, no standard curve was included in the qPCR assay, which limits our ability to formally assess amplification efficiency and to interpret very high Ct values quantitatively; future studies should incorporate a standard curve to allow proper quality control of the assay and clearer interpretation of high-Ct results.

In conclusion, this study provides current epidemiological evidence that urogenital schistosomiasis remains highly prevalent among school-age children in the two peri-urban districts of Lusaka Province, with substantially higher prevalence detected by qPCR than by conventional microscopy. These findings highlight the value of incorporating sensitive molecular diagnostic tools into surveillance programmes and provide contemporary epidemiological data to support targeted mass drug administration and progress towards the WHO goal of schistosomiasis elimination as a public health problem.

## Supporting information

S1 Table Spreadsheet summarizing demographic information and diagnostic indicators for study participants, including district, age, sex, urine dipstick results, *Schistosoma haematobium* egg counts, and test results by microscopy, qPCR, and ELISA. (XLSX)
