# Supplementary material for: Schistosoma haematobium infection among school age children in two peri-urban areas of Lusaka, Zambia: A cross-sectional study
Source: PLoS Negl Trop Dis. 2026 Oct 21;20(10):e0014013. doi: 10.1371/journal.pntd.0014013 (PMC13705608; doi:10.1371/journal.pntd.0014013)
Supplement: Supplementary file 1 [file pntd.0014013.s003.pdf]

Instituto de Salud Carlos III

MINISTERIO
DE CIENCIA, INNOVACIÓN
Y UNIVERSIDADES

31th July 2026

Dear PloS Editors,

REJOINDER: PNTD-D-26-00269

Please find enclosed a revised version of our manuscript entitled "Prevalence and molecular identification of *Schistosoma haematobium* infection in two peri-urban areas of Lusaka, Zambia: a cross-sectional study". We sincerely appreciate the valuable suggestions and comments provided by the referees and the editorial team, which have significantly contributed to improving our study. Below, we address all the concerns raised in a point-by-point manner. We hope that the revised manuscript is now suitable for publication in PLoS Neglected Tropical Diseases.

Journal requirements:

1) Please ensure that the CRediT author contributions listed for every co-author are completed accurately and in full.

RESPONSE: This information has been included.

2) We ask that a manuscript source file is provided at Revision.

RESPONSE: Manuscript with track changes has been uploaded. A clean copy has also been included.

3) We do not publish any copyright or trademark symbols that usually accompany proprietary names, eg ©, ®, or TM (e.g. next to drug or reagent names). Therefore, please remove all instances of trademark/copyright symbols throughout the text, including:
- ® on pages: 7, 10, and 14
- TM on page: 9.

RESPONSE: These symbols have been removed.

4) Thank you for including an Ethics Statement for your study. Please include: i) A statement that formal consent was obtained (must state whether verbal/written) OR the reason consent was not obtained (e.g. anonymity). NOTE: If child participants, the statement must declare that formal consent was obtained from the parent/guardian.].

RESPONSE: This has been amended.

5) Please amend your detailed Financial Disclosure statement. This is published with the article. It must therefore be completed in full sentences and contain the exact wording you wish to be published. 1) State what role the funders took in the study. If the funders had no role in your study, please state: "The funders had no role in study design, data collection and analysis,

decision to publish, or preparation of the manuscript."2) If any authors received a salary from any of your funders, please state which authors and which funders. If you did not receive any funding for this study, please simply state: "The authors received no specific funding for this work."

RESPONSE: This information has now been included.

Reviewer 1:

1) The authors refer to a higher rate of false positives as observed in the ELISA results, though referring to as residual antibodies being persist in serum/urine for several months after praziquantel treatment. This is a known phenomenon the reason ELISAs cannot be used for evaluation of treatment success. Further this provides a complication as post treatment the killed worms and eggs tend to provide as stimulation effect leading to elevated antibodies. The authors need to explain the need for such method use as I the design of the study.

RESPONSE: We thank the reviewer for this pertinent comment. We fully acknowledge that serological assays cannot distinguish between current and past infections. The rationale for including serology in this study was not to evaluate treatment success, but rather to explore whether an apparent absence of parasitologically detectable infection in certain populations reflected a true absence of Schistosoma exposure or, alternatively, a decline in detectable infection due to previous mass drug administration (MDA) activities in the study region. In this context, serology was used as a complementary tool to assess historical exposure at the population level rather than active infection at the individual level. We have now clarified this rationale in the Introduction (lines 147-149) to avoid any potential misunderstanding regarding the intended purpose and interpretation of the serological results.

2) What is the contribution of treatment and that effect of immunizing post MDA? In Kafue without the evidence of MDA there could be contribution of immune response development. Treatment is known to eliminate the infection, but the killed worm are also understood to contribute to evoking a specific immune response.

RESPONSE: We thank the reviewer for raising this point. Please, note that according to the NTD unit at the Ministry of Health, the last countrywide (including Chonwe and Kafue) mass treatment countrywide was conducted in 2020. We agree that both praziquantel treatment and prior MDA history can influence the immune response profile of a population, including antibody titres and the antigenic stimulation that follows the killing of adult worms. We have expanded the Discussion to explicitly address the potential contribution of treatment-induced immune responses to the serological findings, noting that praziquantel-mediated killing of adult worms releases antigens that can stimulate antibody responses and may contribute to partial protective immunity against reinfection. In Kafue, we now note that any elevated antibody responses more plausibly reflect recent anthelminthic treatment received outside a formal MDA programme rather than a distinct MDA-related immunising effect. This clarification has been incorporated into the revised Discussion.

3) The clustering of Kafue and Chongwe sequences requires further explanation in addition to the indication of limited genetic differentiation across these sites. As the transmission may involve spreading across sites in each region and the generation of hybrids. Probably give an explanation to the high gene flow within these endemic areas.

RESPONSE: We thank the reviewer for this comment. In light of this point, and the related concerns raised independently by Reviewers 2 and 3 regarding the robustness of our molecular species/hybrid identification approach (T-ARMS-PCR and ITS-based Sanger sequencing alone, without complementary mitochondrial markers such as cox1), we have removed the phylogenetic analysis and the associated discussion of hybridisation and gene flow between Kafue and Chongwe from the manuscript. We agree that a robust characterisation of gene flow and hybridisation requires combined nuclear and mitochondrial marker analysis and larger sample sizes, which were beyond the scope of the present study. This section, together with the corresponding figure and results paragraph, has therefore been removed, and the manuscript now focuses on the parasitological, serological and qPCR-based diagnostic and prevalence data, which we believe are robust and represent the core contribution of this study.

4) Are the hybrids infectious? This could be a concern and would benefit from additional explanation. The explanation on the hybridization between human and animal schistosome species can give pointers of possible hosts, creating zoonotic reservoirs that sustain transmission even when human treatment coverage is high. This calls for One Health and incorporation of zoonotic NTDs investigation in the control and prevention of schistosomiasis.

RESPONSE: We thank the reviewer for this important and insightful comment. As explained above, and in line with the related concerns raised independently by Reviewers 2 and 3 regarding the robustness of our species/hybrid identification approach (T-ARMS-PCR and ITS sequencing alone, without cox1 or other complementary markers), we have removed the molecular species/hybrid identification component from the manuscript in its entirety, and we are no longer making claims regarding the occurrence, viability or infectivity of S. haematobium × S. curassoni hybrids based on our own data. We fully agree, however, that this is an important issue for the field, and we have added a short paragraph to the Discussion (lines 388-397) noting that, although our study did not include molecular species identification and therefore cannot confirm the occurrence of hybrids in Chongwe or Kafue, hybridisation between human and livestock-associated schistosomes has been increasingly documented elsewhere in sub-Saharan Africa and may create zoonotic reservoirs that sustain transmission even where human treatment coverage is high. We highlight that this underscores the importance of a One Health perspective, including molecular surveillance of livestock and snail intermediate hosts, in future schistosomiasis control and elimination strategies, and we intend to investigate hybrid occurrence and infectivity directly, using appropriate nuclear and mitochondrial markers together with infectivity assays, in a dedicated follow-up study.

5) Specific comments

- Abstract: line 23 the word Sub-Saharan Africa should be written sub-Saharan Africa.
- Line 250: on an ABI
- Line 287: (111 from Chongwe and 97 from Kfaue) correct the spelling of Kafue?

- Line 476: Firstly, the survey was restricted to paediatric populations in only two districts of Lusaka Province. The term paediatric should be used cautiously since the age group considered in this work is not paediatric.

RESPONSE: All suggested comments have been addressed, and the manuscript has been amended accordingly.

Reviewer 2:

The paper presents urogenital infection data and molecular data from eggs from humans. Although the infection data is informative the authors focus too much on the "hybrid" sorry with no robust data. The authors need to be very careful in their data analysis and interpretation related to this subject. The infection data is very useful and informative and I suggest the authors focus more on that. Currently the paper reads like it is lots of parts of studies brought together. All my comments are detailed below rather than be split by sections.

1) ABSTRACT.

- Revise the abstract and summary according to the comments on the paper.

RESPONSE: We thank the reviewer for this comment. The Abstract and Author Summary have been revised to reflect the removal of the molecular species/hybrid identification component (see our responses below) and now focus on the parasitological, serological and qPCR-based prevalence data, which represent the robust, core findings of the study. All references to hybrid Schistosoma forms have been removed from the Abstract and Author Summary, and district-level prevalence data have been added, as also requested by Reviewer 3 (see below).

2) INTRODUCTION

- Line 107: You need to explain the need for serological detection as it does not distinguish present and past infections so its use is limited.

RESPONSE: This comment has also be raised by Reviewer 1, and we have amended the introduction accordingly (lines 147-149).

3) METHODS

- Line 123: You need to explain what the further action was for those infected.

RESPONSE: All infected participants were treated with praziquantel (40mg/kg body weight) according to national treatment guidelines. This information has now been included in lines 168-169.

- Line 141: "and later are infected" – they are infected at the time of water contact so revise.

RESPONSE: This has been corrected.

- Line 143: add the details of what the systematic random sampling method is.

RESPONSE: This information has now been included in lines 188-199.

- Line 150: you do not explain how the microscopic leucocytes, biochemistry protein parameters were measured.

RESPONSE: This information has now been included in lines 204-207.

- Line 156: were the eggs not stained?

RESPONSE: No, eggs were visible in the filter without the need for staining.

- Lines 159-180: this is a large area of work. Was this done in Zambia and add details as to why this method was used versus an available kit.

RESPONSE: Recombinant protein production and the associated molecular and serological analyses were performed in Spain. This work was supported through a Women for Africa Foundation fellowship awarded to the first author (M.M. Mutengo), which enabled the author to perform this work in collaboration with our lab in Spain. To the best of our knowledge, there is currently no commercially available serological kit specifically validated for S. haematobium, and existing assays targeting schistosomiasis often show limited specificity due to cross-reactivity. The recombinant antigen used in this study has demonstrated superior performance compared with other available serological approaches, particularly in terms of sensitivity and specificity, as previously reported [1].

- Line 210: In the results you do not mention what the Nanodrop informed you about your samples. Also, bear in mind the Nanodrop will measure any DNA and this includes human DNA so the quantification is not specific.

RESPONSE: We agree that Nanodrop measurements do not provide information on the biological origin of extracted DNA. However, Nanodrop spectrophotometry is commonly used following DNA extraction to estimate DNA concentration and assess sample purity, including the presence of potential contaminants such as phenol or other extraction reagents. We have clarified this point in the Methods section to ensure that Nanodrop values are interpreted solely as a quality and quantification control step and not as an indicator of DNA origin or integrity.

- Line 223: What part of the urine were these taken from and how captured (it is not easy to find individual eggs and capture them. You say above that the urine was processed by microscopy and for molecular so was there urine left? Also, explain the procedure for the extraction or how is was adapted to eggs.

RESPONSE: We thank the reviewer for this comment and appreciate the opportunity to clarify the procedure. A minimum urine volume of 5 ml was collected from each participant. Of this, 4 ml were processed for molecular analysis, while the remaining 1 ml was reserved specifically for egg isolation. We have removed the sections regarding the molecular characterisation from the manuscript, so we believe this information is no longer needed.

- Lines 221-243: The T-ARMS description is fine but include all the information on the amplicon in a figure as it is not information in the main text.

RESPONSE: We have removed the sections regarding the molecular characterisation from the manuscript, so we believe this information is no longer needed.

- Line 247: Why these bands? It is not clear how you would identify the SNP's from these bands? Also, your NCBI accessions are all 518 bp long so how does that work with sequencing bands of only 487 and 234?

RESPONSE: We have removed the sections regarding the molecular characterisation from the manuscript, so we believe this information is no longer needed.

- Line 253-255: You have to be very careful when BLASTing ITS sequences for Schisto species identification as the output is very often misinterpreted. It is better just to look at the species specific SNPs'.

RESPONSE: We have removed the sections regarding the molecular characterisation from the manuscript, so we believe this information is no longer needed.

- Line 258-269: Running a phylogenetic analysis on ITS data for Sb, Sh and Sc is not informative due to the high level of conservation. It does not tell you anything more than what looking at the specific specific SNP's tell you.

RESPONSE: As indicated above, the phylogenetic analysis based on ITS sequences has been removed from the manuscript. We agree that, given the high level of conservation of this marker among S. haematobium-group species, this analysis added limited value beyond what species-specific SNP genotyping would provide.

- Line 256: See comments below on the sequences. The naming of the sequences on NCBI must be changed to reflect the incorrect assignment to Sh/Sc.

RESPONSE: We thank the reviewer for this comment. As this analysis has been removed from the manuscript, we will also review and, where necessary, amend or withdraw the associated GenBank accessions outside of the manuscript submission process, to ensure that no incorrect Sh/Sc species assignments remain publicly associated with our sequences.

- Line 261: When you are using ITS data from NCBI that is labelled as Sh/Sc or Sh/Sb you have to think about what that data is from and how informative it is. For example ITS data labelled Sh/Sb maybe from what is assumed to be a Sh/Sb hybrid that has a cox1 Sb and an ITS Sh so the ITS is just Sh and no different to a pure Sh. This is a common mistake many researcher make and just automatically assign data as from a hybrid because of the blast hit but if you look the BLAST hit will be 100% for the "hybrid" and for Sh. You must look at the data, think about it, think about what it is telling you and interpret it. All BLAST searches must be analysed informatively and not just be taken as whatever is at the top of the list.

RESPONSE: We agree with this important observation and thank the reviewer for the detailed explanation. As noted above, we have removed the molecular species/hybrid identification section from the manuscript and no longer make claims regarding hybrid status based on ITS/BLAST data alone.

4) RESULTS

- Line 299: why are leukocytes informative for your study?

RESPONSE: We thank the reviewer for this insightful comment. Urinary leukocyte detection was included as an indicator of urinary tract inflammation, which may be associated with Schistosoma haematobium infection but can also reflect co-existing conditions such as bacterial urinary tract infections. Reporting leukocyturia therefore provides important contextual information for interpreting haematuria results in endemic settings. Importantly, recent evidence supports the relevance of leukocytes in active egg-producing schistosomiasis, with increased leukocyte infiltration (including eosinophils and neutrophils) reported in individuals with ongoing parasite egg excretion [2].

In our study, leukocyte positivity was not used as a diagnostic criterion for schistosomiasis but rather as supportive information to contextualize urine reagent strip findings. The partial overlap observed between leukocyturia and haematuria further supports the inclusion of leukocyte data as complementary rather than redundant information. While visible haematuria was detected in 10.6% of participants, leukocytes were present in a larger proportion, and only 29 of the 40 leukocyte-positive individuals also exhibited microhaematuria. This suggests that leukocyturia may reflect inflammatory or immune-mediated responses that are not necessarily accompanied by detectable bleeding at the time of sampling.

We have clarified this interpretation in the revised manuscript and emphasize that leukocyte data were included to aid interpretation of haematuria patterns and underlying urinary tract pathology, rather than as a marker of infection per se (lines 377-379).

- Line 306-308, 319-320: do not repeat methods in the results

RESPONSE: We thank the reviewer for this comment. We have revised the Results section to remove methodological detail that duplicated the Methods section, retaining only the information necessary to interpret the reported findings.

- Line 326-330: it is not clear what the ELISA data adds? Also, there is a large section on this in the methods but a very small bit in the results?

RESPONSE: We thank the reviewer for this comment. We have revised the Results to present the ELISA/serology findings with a level of detail comparable to that given for microscopy and qPCR. We also clarify in the text that serology was included to provide population-level information on prior exposure to Schistosoma spp., complementing the individual-level infection data obtained by microscopy and qPCR, which explains the more limited methodological detail required in the Results relative to the Methods.

- Line 332: you mention correlation to age on 3.1 so you might want to change the structure.

RESPONSE: We thank the reviewer for this observation. We have restructured the Results so that all age-related associations, both pooled and by district, are presented together within a single, clearly labelled subsection, avoiding the previous fragmentation of age-related findings across different parts of the Results.

- Lines 332-356: It is really not clear what your data is saying as you combine all diagnostics and age and sex and region. Consider restricting this and think about how the data can be informative – are you comparing diagnostics or are you comparing infection levels (age, sex, region?).

RESPONSE: We thank the reviewer for this important comment. We have restructured the Results to more clearly separate (i) the comparison of diagnostic methods (microscopy, ELISA and qPCR) from (ii) the analysis of infection levels by age, sex and district. Each diagnostic method is now presented with its own prevalence estimates by sex and district, followed by a dedicated paragraph on the association between age and infection status, to improve clarity and avoid conflating these two distinct types of comparison.

- Line 363: There is no text in your introduction to mention the "hybrid" topic. So many researcher now bring this into the studies without much thought of why or what it means. If you are going to present data on this topic you need to read the background literature, think about the meaning and make informed analysis of the data. You cannot make big statements without robust data.

RESPONSE: We thank the reviewer for this comment. As the molecular species/hybrid identification component has been removed from the manuscript (see responses above), the "hybrid" topic is no longer discussed, and this comment is now resolved.

- Lines 361-381: There are major issues with this data and the way it has been analysed and interpreted. These are listed below.

RESPONSE: We thank the reviewer for this detailed assessment. In response to this comment and the specific points raised below, we have removed the molecular species/hybrid identification section (T-ARMS-PCR, Sanger sequencing and the associated phylogenetic analysis) from the manuscript in its entirety, together with the corresponding figure and results paragraph. We agree that, without complementary mitochondrial markers, a larger sample size and a more rigorous SNP-based analysis, this dataset did not support robust conclusions regarding hybridisation, and we preferred to remove it rather than risk presenting an overinterpreted dataset.

- You do not include how many eggs from each participant were analysed.

RESPONSE: As this section has been removed from the manuscript (see above), this comment is now resolved. We thank the reviewer for highlighting the need for this information and will ensure it is reported in full detail in any future, dedicated molecular characterisation study.

- There is no cox1 data associated with these samples so you cannot make any robust inferences on species or "hybrid". Be aware there is many papers now showing the the use of these basic molecular markers e.g. ITS are not robust for "hybrid" identification in terms of what a "hybrid" actually is and the biological consequences.

RESPONSE: We fully agree with the reviewer. The absence of cox1 (or other complementary marker) data is indeed a critical limitation for robust hybrid identification and was a central reason for our decision to remove the molecular species/hybrid identification section from the manuscript.

- On your gel figure it is not clear what the samples represent and what did you use as your controls?

RESPONSE: As the T-ARMS-PCR gel figure (previous Supplementary Figure 1) has been removed from the manuscript along with the associated molecular identification section, this comment is now resolved.

- You phylogenetic analysis is not informative and see the notes above on using ITS data from NCBI and BLAST. You can see that there is no structuring for Sb, Sc. You do have some structuring for Sh and in fact that shows that if anything your samples are Sh.

RESPONSE: We thank the reviewer for this detailed analysis of our data, which we found very helpful. We agree with this assessment and have removed the phylogenetic analysis (previous Figure 2), together with the associated interpretation regarding hybridisation, from the manuscript.

- Line 374 – 375 – that is a strange statement as why would you expect them to cluster? The authors should think about what they are trying to understand by the data analysis.

RESPONSE: As this section of the manuscript has been removed (see above), this specific statement no longer appears in the revised manuscript.

- In this section you do not mention anything about the S. curassoni / Sh that you mention elsewhere in the paper and that you have put as an ID for some of your sequences. This is very strange.

RESPONSE: As the molecular species/hybrid identification section, including all references to S. curassoni and hybrid designations, has been removed from the manuscript, this inconsistency no longer applies.

- All you sequences on Genbank are Sh ITS data. You must change the accessions that say they are Sh/Sc as they are not correct and will mislead other researcher.

RESPONSE: We thank the reviewer for this important observation. Alongside the removal of the molecular identification section from the manuscript, we will review and, where necessary,

amend or withdraw the associated GenBank accessions to ensure they do not misrepresent the sequences as Sh/Sc when they are more consistent with Sh ITS data alone.

- The authors should think more about species and geographical locations. S. curassoni is only found in the West of Africa due to the specific snail it uses for transmission. It is not understood why the authors would identify data within their study as being S. curassoni without questioning it. they should also mention that the T-ARMS PCR has obviously given an in-correct profile for Sh.

RESPONSE: We thank the reviewer for this valuable clarification regarding the biogeography of S. curassoni and its snail intermediate host. We agree that assigning an S. curassoni identity to samples from Zambia without further scrutiny was not appropriate, and that the T-ARMS-PCR assay most likely produced a misleading profile in this context. This is a further reason why we have removed the molecular species/hybrid identification section from the manuscript.

- Notes on your sequences:
    - They are all Sh ITS
    - Some of your sequences contain missing bp which is why your BLAST searches may have mislead you.
    - They are longer than the fragments that you cut out of the gels.

RESPONSE: We thank the reviewer for this careful re-analysis of our sequence data, which we found extremely helpful. We agree with these observations and, in light of them, have removed the molecular species/hybrid identification section, the associated sequence-based claims, and Figure 2 from the manuscript in their entirety.

- It is not clear how the molecular ID relates to the study as it is mainly about diagnosis and prevalence levels. You do not need to just bring in molecular identifications as an add on especially if there is no hypothesis or robust methods.

RESPONSE: We fully agree with the reviewer. On reflection, we recognise that the molecular species/hybrid identification data were not sufficiently hypothesis-driven or methodologically robust to be included alongside our diagnostic/prevalence dataset, and risked distracting from the core findings of the study. We have therefore removed this component entirely, and the manuscript now focuses on the prevalence of urogenital schistosomiasis and the comparative performance of microscopy, ELISA and qPCR as diagnostic tools.

5) DISCUSSION

- Lines 384-400: This is introduction and not discussion

RESPONSE: We thank the reviewer for this comment. We have revised the opening of the Discussion to remove background information that duplicated the Introduction.

- Lines 407-416: This is why it is not clear why the ELISA was done. Also, these are not false positives. They are positive for antibodies due to past infection and it is not a phenomenon?

RESPONSE: We thank the reviewer for this important clarification, and we agree with the underlying point. We have revised the Discussion to avoid describing ELISA-positive results as 'false positives', since these most likely reflect antibodies from past infection or previous treatment rather than current active infection. The rationale for including serology, namely to provide population-level information on historical exposure rather than to diagnose current infection, is now clarified both in the Introduction (lines 106–109) and in the Discussion.

- Line 446-447: This is a very bold statement with not robust data and you do not detail that in the results.

RESPONSE: We thank the reviewer for this comment. We have removed the phylogenetic analysis and the associated discussion of hybridization from the manuscript.

- Line 449: The reference 12 also has mis identification of samples reported in it. There is no S. bovis in the data. They also only looked at ITS. Have a look at the data. As academics you should always look at the detail associated with unusual findings.

RESPONSE: We appreciate the reviewer's comment, and, based on this and other comments from other reviewers, we have removed the molecular species/hybrid identification section.

- Line 450-455: Do you think your data is robust enough to state this?

RESPONSE: We agree with the reviewer's concern. The corresponding statements, which relied on the now-removed molecular species/hybrid identification data, have been removed from the Discussion.

- Revise the rest of the discussion based on comments above.

RESPONSE: We thank the reviewer for this overarching comment. The Discussion has been thoroughly revised throughout, removing all statements relating to hybrid Schistosoma species/molecular identification (in line with the removal of this section from the Results), and now focuses on the interpretation of the prevalence and diagnostic performance data obtained by microscopy, ELISA and qPCR.

Reviewer 3:

1) Abstract: Under the results sub-heading, prevalence in each district should be stated.

RESPONSE: We thank the reviewer for this suggestion. We have amended the Abstract to state the prevalence of urogenital schistosomiasis observed in each district (Chongwe and Kafue) by microscopy and qPCR.

2) Abstract: Under the results sub-heading, the authors report on associations between S. haematobium infection and age + sex, but then clarify in the next sentence that many eggs were hybrids. Therefore, I suggest changing the original statement to say that urogenital schistosomiasis (not S. haematobium specifically) was associated with age.

RESPONSE: We thank the reviewer for this suggestion. As the molecular species/hybrid identification component has been removed from the manuscript, the Abstract and manuscript now consistently refer to 'urogenital schistosomiasis' and to 'S. haematobium infection' as diagnosed by microscopy, ELISA and qPCR, without reference to hybrid forms, which resolves this inconsistency.

3) Introduction, lines 88-91 and 103-105 state historical prevalence estimates, including from Lusaka. Please clarify when estimates were from, as this will help set the scene for the current investigation.

RESPONSE: We thank the reviewer for this comment. We have amended the Introduction to specify the approximate years in which the cited historical prevalence estimates were obtained, to better contextualise the current investigation relative to previous surveys.

4) Some analysis of infection intensity and comparison between sites would be useful.

RESPONSE: We thank the reviewer for this suggestion. This comparison is already reported in the manuscript: the median number of eggs per 10 ml of urine was compared between microscopy-positive participants from Chongwe and Kafue using a two-tailed Mann-Whitney test (Results, section 3.2), and no statistically significant difference was found between districts. We have revised the text to make this comparison easier for readers to locate.

5) An additional results table assessing diagnostic performance (sensitivity, specificity, PPV, NPV of microscopy and ELISA vs qPCR) would be welcome.

RESPONSE: We thank the reviewer for this valuable suggestion. We have added a new table (Table 4) reporting the sensitivity, specificity, PPV and NPV of all four index tests relative to a composite reference standard. We have included a new section in Materials and Methods and in Results to include all this information.

6) The study area and population is clearly described. However, insufficient information about the sampling design is provided. A sample size calculation should be stated, with reasoning.

RESPONSE: We thank the reviewer for this comment. We have added a description of the sample size calculation to the Methods, based on an expected prevalence derived from previous studies in similar peri-urban settings in Lusaka Province, a 95% confidence level and a 5% margin of error. (lines 188-199).

7) Diagnostic procedures upon urine samples are generally well described, but I have the following suggestions: specify the Ct cut-off for positivity in the qPCR assay, confirm if an internal amplification control was used (if not, recognise that this may lead to false negative results).

RESPONSE: We thank the reviewer for these comments. We have clarified in the Methods that samples were considered positive when amplification occurred with a Ct value ≤ 40 cycles. The Homo sapiens 18S gene was targeted as an internal amplification control multiplexed in qPCR assay.

8) I have some concerns about the molecular characterisation of eggs. 64 individual eggs from 24 patients were analysed, and only 29 egg samples were subjected to ITS sequencing. Please clarify how the 24 patients and the 29 sequenced egg samples were selected. How many eggs were analysed per patient and why? How might this impact inferences? Given the restricted species scope of the T-ARMS-PCR assay, might some other species have been missed that are known to hybridise with S. haematobium in Zambia and neighbouring countries (e.g. S. mattheei). Please clarify was this assay chosen over assays that can detect more species in the S. haem group. Expansion on these topics is needed in the limitations section.

RESPONSE: As the molecular species/hybrid identification component, including the T-ARMS-PCR-based analysis of individual eggs, has been removed from the manuscript, this comment no longer applies to the revised version. We thank the reviewer for these important considerations, including the possible role of additional species such as S. mattheei, which we will take into account in the design of future, dedicated molecular studies.

9) Demographics paragraph and Table 1: reporting mean age with CIs is a bit unconventional for descriptive demographics. Mean + SD or median with IQR would be more typical.

RESPONSE: We thank the reviewer for this comment. We have amended Table 1 and the corresponding text in the Results to report mean age with standard deviation (mean ± SD) rather than 95% confidence intervals, in line with conventional reporting for descriptive demographic data.

10) Figure resolution is poor but this may be just an issue with the PDF sent to me for review.

RESPONSE: We thank the reviewer for this comment. We believe all figures had the appropriate resolution. For resubmission, we have re-exported all figures at 300 dpi and TIFF format to avoid any quality loss during PDF conversion.

11) Figure 2 provides useful species-level context, but ITS-based phylogeny is limited. The tree presentation could be improved to include colour-based annotations to show location and species, which would make its interpretation easier for the reader.

RESPONSE: As Figure 2 (the ITS-based phylogenetic tree) has been removed from the manuscript, following the concerns raised above regarding the molecular species/hybrid identification analysis, this comment no longer applies.

12) Discussion lines 401-402 comment on diagnostic sensitivity and specificity but these are not presented in the results section

RESPONSE: We thank the reviewer for this comment. This part of the discussion has been amended to say "prevalence" instead of "sensitivity". Furthermore, as noted in our response to comment 5 above, we have added a new results table (Table 4) reporting the sensitivity, specificity, PPV and NPV of all four index tests relative to a CRS, as detailed in our response to comment 5 above.

13) Molecular work shows that hybrids are present, but the limitations of the T-ARMS-PCR assay and ITS-based phylogeny prevent broader population-level conclusions

RESPONSE: As the T-ARMS-PCR and ITS-based phylogenetic analyses have been removed from the manuscript, this comment no longer applies to the revised version.

14) The limitations surrounding the molecular characterisation of eggs needs more discussion (see earlier comment in methods).

RESPONSE: As the molecular characterisation of eggs has been removed from the manuscript, the corresponding limitations no longer need to be discussed. We thank the reviewer for these observations, which will be taken into account in future work.

15) The findings are clearly framed in the context of schistosomiasis control and elimination efforts.

RESPONSE: We thank the reviewer for this positive comment; no changes were required.

16) There are some minor typographical errors: "Kfaue" should be corrected to "Kafue", "sensitivity" instead of "sensibility".

RESPONSE: These typos have been corrected.

17) Improved presentation of Figure 2 is recommended (as above).

RESPONSE:Figure 2 has been removed from the manuscript.

<u>REFERENCES</u>:

1. Pearson MS, Tedla BA, Mekonnen GG, Proietti C, Becker L, Nakajima R, et al. Immunomics-guided discovery of serum and urine antibodies for diagnosing urogenital schistosomiasis: a biomarker identification study. Lancet Microbe. 2021;2: e617–e626. doi:10.1016/S2666-5247(21)00150-6

2. Schnyder JL, Gobbi F, Schunk M, Lindner A, Salvador F, Duvignaud A, et al. Can haematological changes constitute a surrogate diagnostic parameter to detect schistosomiasis in migrants and travellers? - A retrospective analysis. New Microbes New Infect. 2023;53: 101136. doi:10.1016/j.nmni.2023.101136

We hope it now meets the requirements for publication at *PLoS Neglected Tropical Diseases* and look forward to hearing from you.

Yours sincerely,

Javier Sotillo,

On behalf of all authors.
