# Supplementary material for: Schistosoma haematobium infection among school age children in two peri-urban areas of Lusaka, Zambia: A cross-sectional study
Source: PLoS Negl Trop Dis. 2026 Oct 21;20(10):e0014013. doi: 10.1371/journal.pntd.0014013 (PMC13705608; doi:10.1371/journal.pntd.0014013)
Supplement: Supplementary file 2 [file pntd.0014013.s004.docx]

25^th^ August 2026

**Dear PloS Editors,**

REJOINDER: PNTD-D-26-00269

Please find enclosed a revised version of our manuscript entitled “Prevalence and molecular identification of *Schistosoma haematobium* infection in two peri-urban areas of Lusaka, Zambia: a cross-sectional study”. We sincerely appreciate the valuable suggestions and comments provided by the referees and the editorial team, which have significantly contributed to improving our study. Below, we address all the concerns raised in a point-by-point manner. We hope that the revised manuscript is now suitable for publication in PLoS Neglected Tropical Diseases.

**Reviewer 1:**

1) Please provide the GPS locations of the study areas.

*RESPONSE: We thank the reviewer for this request. We have added the GPS coordinates of the four participating schools to the Methods (Study area and population): Evergreen Primary School (15.2672°S, 28.64602°E) and Shiyala Primary School (15.2921°S, 28.66161°E) in Chongwe District; Chanyanya Primary School (15.6535°S, 28.03168°E) and Mungu Primary School (15.6971°S, 28.11493°E) in Kafue District.*

2) The statement requires elaboration as to the reason ELISA results are as indicator of cumulative population-level exposure to Schistosoma spp. rather than a direct measure of current infection status. How is this explained where ELISA is believed for distinguishing past from current exposure? What does cumulative mean?

*RESPONSE: We thank the reviewer for asking us to clarify this point. IgG antibodies against the Sh-TSP-2 antigen are generated in response to any exposure to Schistosoma spp. antigens, whether from a current, ongoing infection or a previous infection that has since been cleared (spontaneously or following treatment). Because these antibodies can persist for many months to years after the parasite itself is no longer present, a positive ELISA result reflects the cumulative history of exposure to the parasite over time, rather than the presence of a currently patent infection. In other words, ELISA cannot distinguish whether a positive signal arises from an infection acquired last week or one cleared two years ago; both contribute to the same measurable antibody response. We have revised the manuscript to state this explicitly rather than using the term "cumulative" without definition.*

3) A clearer explanation should be made in the discussion on the hierarch of diagnostic methods: microscopy, ELISA, and qPCR - high analytical sensitivity for detecting parasite DNA, qPCR showed the highest sensitivity (Table 4).

*RESPONSE: We thank the reviewer for this suggestion. We have added a sentence to the Discussion explicitly stating the expected hierarchy of analytical sensitivity among the three diagnostic approaches used in this study: microscopy < ELISA < qPCR. This hierarchy reflects the fact that microscopy requires the presence of intact, excreted eggs, ELISA detects antibody responses that develop only after a sufficient antigenic stimulus, whereas qPCR can detect trace amounts of parasite DNA independently of egg excretion, giving it the highest analytical sensitivity of the three methods, as reflected in our sensitivity estimates against the composite reference standard (Table 4).*

**Reviewer 2:**

1) Line 146-155 – It is not very clear how the randomisation and random sampling were done. Maybe some references could support the statements. Also, line 155/6 – what do you mean by sampling interval?

*RESPONSE: We thank the reviewer for this comment. We have clarified the systematic random sampling procedure and formally defined the sampling interval (k) as the ratio of the number of eligible children present at each school (N) to the required sample size (n): following a randomly chosen starting point, every k^th^ eligible child was selected until the required sample size was reached. We have also added a supporting reference from a comparable school-based schistosomiasis study using the same approach* [1]*.*

2) Line 172 – The production of the ELISA reagents should be mentioned in the introduction, and more explanation of why this was done should be included, e.g. that previous studies have shown that it is better than other methods such as SEA. This part should also come under a general heading of ELISA. It is not clear why this method has not be accepted more widely. For example, ELISA is used as a diagnostic for traveller's infections, etc., and SEA is used.

*RESPONSE: We thank the reviewer for this suggestion. We have added a sentence to the Introduction noting that a recombinant S. haematobium antigen (Sh-TSP-2) was produced and used to develop an in-house ELISA for this study, rather than the more traditional soluble egg antigen (SEA). We now briefly explain the rationale: SEA is a crude, poorly standardised antigen preparation that is well documented to cross-react with other helminth infections, and is costly and difficult to produce in a standardised manner across laboratories, whereas defined recombinant antigens such as Sh-TSP-2 allow for a consistent, reproducible reagent with improved specificity* [2]*. We have also merged the "Recombinant protein production" and "ELISA assays" subsections under a single "ELISA" heading, as suggested. Regarding why ELISA has not been more widely adopted in endemic-country field surveys despite its established use in travel medicine clinics (where it is valued for detecting light, early infections that microscopy would miss): we note in the Discussion that this largely reflects practical barriers (i.e. the cost and technical capacity required to produce and validate a recombinant antigen, the absence of a widely available standardised commercial assay for African populations, and the inherent inability of any antibody-based test to distinguish active from past infection, which limits its use for prevalence surveys aiming to estimate current transmission).*

3) Line 230 – the inclusion of an internal control has been added, but no optimisation is stated, and no references are given. Typically, very low levels of human DNA are detected in urine. The authors should show some amplification curves for the assay. Additionally, no standard curve is used for the qPCR, and this should be discussed in the paper. It allows quality control of the qPCR and evaluation of what the very high CT values represent, especially as CT values above 35 should be evaluated carefully.

*RESPONSE: We thank the reviewer for this comment. Formal titration/optimisation experiments for the 18S internal amplification control (IAC) were not performed specifically for this study; rather, this IAC is used routinely in our laboratory at the Instituto de Salud Carlos III (ISCIII) for both urine and blood/serum samples as part of our standard diagnostic and research workflow, and its performance is therefore well established through routine use rather than through study-specific optimisation. A representative amplification plot illustrating consistent 18S amplification across representative samples from this study is provided below for the reviewer's consideration:*

*We would also like to clarify that human genomic DNA is reliably and consistently detectable in urine under normal physiological conditions, reflecting the continuous exfoliation of urothelial cells into urine. In individuals with urogenital schistosomiasis, urothelial damage is expected to further increase the shedding of human cells (and therefore human DNA) into urine, making failure of the 18S IAC due to insufficient human DNA input particularly unlikely in this population. Regarding the absence of a standard curve, we agree this limits our ability to formally assess amplification efficiency and to interpret very high Ct values quantitatively; we have added this explicitly as a limitation in the Discussion, together with a recommendation that future studies incorporate a standard curve to allow proper quality control of the assay and clearer interpretation of high-Ct results.*

4) Line 256 – should ELISA be included in the composite reference, as it cannot distinguish between active and past infection and therefore cannot be compared to the other tests?

*RESPONSE: We thank the reviewer for raising this important methodological point. We elected to retain ELISA as one of the four index tests contributing to the CRS, following a similar approach previously applied to schistosomiasis diagnostics by Beltrame et al. (2017)* [3]*, who likewise incorporated serology alongside parasitological and molecular tests when constructing a composite reference standard in the absence of a true gold standard. We would like to clarify that, by definition, a positive ELISA result alone cannot render a participant CRS-positive: participants were classified as infected by the CRS only if they had a positive microscopy result or, among microscopy-negative participants, if at least two of the remaining three index tests (microhaematuria, ELISA, qPCR) were concordantly positive. ELISA therefore only contributes to a CRS-positive classification when its result is concordant with at least one other index test. We nonetheless acknowledge that, because ELISA cannot distinguish between past and current infection, its concordance with another positive test does not guarantee that the underlying infection is currently active, and this is added as a limitation when interpreting the CRS-based diagnostic performance estimates..*

5) Some intensity data should be brought into the results based on CT values and egg counts. This would be very informative in comparison to morbidity assessments, e.g. haematuria, etc. If this data is not available, then you should state this in the methods, and that the data were treated as binary, e.g. positive and negative. CT values are needed to assess the assay’s potential specificity.

*RESPONSE: We thank the reviewer for this suggestion. Ct values were not systematically recorded as a continuous variable for all samples in this study; qPCR results were treated as binary (positive/negative) based on the pre-specified Ct ≤ 40 cut-off. We have added this explicitly to the Methods (Statistical Analysis) to clarify that intensity-based comparisons between qPCR and egg counts were not performed.*

6) Line 351 – the authors should discuss what trace amounts of DNA would mean in terms of an infection, as it would not be eggs?

*RESPONSE: We thank the reviewer for asking us to clarify this. By "trace amounts of parasite DNA" we do not mean eggs, but rather cell-free or fragmented Schistosoma genomic DNA that can be present in urine independently of visible, intact eggs. Because qPCR amplifies short target sequences rather than requiring an intact, morphologically identifiable egg, it can detect infection even when egg output is too low, or too irregular, to be captured by urine filtration microscopy. We have clarified this distinction in the Discussion.*

7) The ELISA data needs some thought: although it detects past infections, it should also detect current infections, which is what qPCR also detects. You could also discuss the possible false positives for qPCR at very high CT. Most studies implement a CT cutoff of at least 35 due to false positives.

*RESPONSE: We thank the reviewer for this comment. We used a Ct ≤ 40 cut-off for a positive qPCR result. As a quality-control measure, samples yielding Ct values above 35 were subjected to more detailed scrutiny and were repeated to confirm the amplification signal before being reported as positive. We have added this clarification to the Methods, and have also added a sentence to the Discussion acknowledging that, in the absence of a standard curve, we cannot fully rule out that some qPCR-positive results in this higher Ct range reflect trace or non-specific amplification rather than robust evidence of active infection (a consideration consistent with the comparatively lower specificity of qPCR observed against the composite reference standard (Table 4)).*

8) Line 430 – if you are going to mention hybrids, you should define what you mean by hybrids and also look at the balanced literature that shows most hybrids in West Africa are ancient forms and do not constitute a zoonotic risk. For Zambia, you should talk about the S. mattheei issue rather than S. bovis and S. curassoni, which are not present there. You should state that there are many unknowns about the zoonotic risk. Very few animals, if any, have been found to be infected with S. haematobium and/or hybrids.

*RESPONSE: Although the present study did not include molecular species identification and therefore cannot confirm the occurrence of Schistosoma hybrids in Chongwe or Kafue, we acknowledge the reviewer's important point that the current literature on Schistosoma hybridisation requires careful, balanced interpretation. We define hybrids here as offspring resulting from interbreeding between S. haematobium and closely related species within the S. haematobium group, which can arise both through recent, ongoing hybridisation events and through ancient introgression whose genomic signatures persist in present-day populations without necessarily reflecting active, contemporary zoonotic transmission* [4]*. Indeed, large-scale genomic analyses of West African S. haematobium populations have found evidence of ancient introgression from S. bovis but no clear signal of ongoing hybridisation, cautioning against interpreting the presence of hybrid genetic markers alone as evidence of an active zoonotic reservoir. For Zambia specifically, S. bovis and S. curassoni are not known to occur, and the livestock-associated species of greatest relevance in this region is S. mattheei, which has been confirmed to hybridise with S. haematobium in neighbouring Malawi, with limited but growing evidence of zoonotic spill-over from cattle* [5]*. However, very few animals in the region have been confirmed infected with S. haematobium or S. haematobium-mattheei hybrids, and substantial uncertainty remains regarding the extent, direction, and epidemiological significance of zoonotic transmission in this part of southern Africa. We have revised the manuscript accordingly, replacing references to S. bovis/S. curassoni with S. mattheei, and explicitly noting these unknowns rather than presenting hybridisation as an established zoonotic risk*

9) Overall, the discussion could be reduced to make it more concise and focused on the data presented.

*RESPONSE: We thank the reviewer for this suggestion. We agree that conciseness and a clear focus on the data presented are valuable, and in this revision we removed a duplicated sentence on treatment-induced immune responses and a speculative sentence on age-related water contact behaviour that was not directly supported by our data. However, in responding to the detailed clarifications requested elsewhere in this review (including the rationale for including ELISA in the composite reference standard, an explicit statement of the expected diagnostic sensitivity hierarchy, clarification of what qPCR-detected "trace amounts" of DNA represent, and a substantially revised, more cautious discussion of Schistosoma hybridisation) additional content was necessarily added to the Discussion. As a result, the Discussion is somewhat longer overall than in the previous version, despite these targeted cuts. We believe this additional detail was necessary to fully and transparently address the other specific points raised by the reviewers and remain happy to trim further if particular sections are felt to be more detailed than needed.*

**REFERENCES**

1.	Muhumuza S, Olsen A, Katahoire A, Nuwaha F. Uptake of Preventive Treatment for Intestinal Schistosomiasis among School Children in Jinja District, Uganda: A Cross Sectional Study. Speybroeck N, editor. PLoS ONE. 2013;8: e63438. doi:10.1371/journal.pone.0063438

2.	Pearson MS, Tedla BA, Mekonnen GG, Proietti C, Becker L, Nakajima R, et al. Immunomics-guided discovery of serum and urine antibodies for diagnosing urogenital schistosomiasis: a biomarker identification study. Lancet Microbe. 2021;2: e617–e626. doi:10.1016/S2666-5247(21)00150-6

3.	Beltrame A, Guerriero M, Angheben A, Gobbi F, Requena-Mendez A, Zammarchi L, et al. Accuracy of parasitological and immunological tests for the screening of human schistosomiasis in immigrants and refugees from African countries: An approach with Latent Class Analysis. Keiser J, editor. PLoS Negl Trop Dis. 2017;11: e0005593. doi:10.1371/journal.pntd.0005593

4.	Webster B, Huyse T. Not all ‘hybrids’ matter: towards a nuanced understanding of *Schistosoma* species hybridization. Philos Trans B. 2026;381: 20240526. doi:10.1098/rstb.2024.0526

5.	Juhász A, Makaula P, Cunningham LJ, Jones S, Archer J, Lally D, et al. Revealing bovine schistosomiasis in Malawi: Connecting human and hybrid schistosomes within cattle. One Health. 2024;19: 100761. doi:10.1016/j.onehlt.2024.100761

We hope it now meets the requirements for publication at *PLoS Neglected Tropical Diseases* and look forward to hearing from you.

Yours sincerely,

Javier Sotillo,

On behalf of all authors.
