# Supplementary material for: Schistosoma haematobium infection among school age children in two peri-urban areas of Lusaka, Zambia: A cross-sectional study
Source: PLoS Negl Trop Dis. 2026 Oct 21;20(10):e0014013. doi: 10.1371/journal.pntd.0014013 (PMC13705608; doi:10.1371/journal.pntd.0014013)
Supplement: Peer review history

# Peer review: Decision Letter 0

**DOI:** 10.1371/journal.pntd.0014013.r001

**Authors:** David J. Diemert, Gabriel Rinaldi

26 Mar 2026

PNTD-D-26-00269

Prevalence and molecular identification of Schistosoma haematobium infection in twoperi-urban areas of Lusaka, Zambia: a cross-sectional study

PLOS Neglected Tropical Diseases

Dear Dr. Sotillo,

Thank you for submitting your manuscript to PLOS Neglected Tropical Diseases. After careful consideration, we feel that it has merit but does not fully meet PLOS Neglected Tropical Diseases's publication criteria as it currently stands. Therefore, we invite you to submit a revised version of the manuscript that addresses the points raised during the review process.

Please submit your revised manuscript within by May 25 2026 11:59PM. If you will need more time than this to complete your revisions, please reply to this message or contact the journal office at plosntds@plos.org. When you're ready to submit your revision, log on to https://www.editorialmanager.com/pntd/ and select the 'Submissions Needing Revision' folder to locate your manuscript file.

Please include the following items when submitting your revised manuscript:

* A letter that responds to each point raised by the editor and reviewer(s). You should upload this letter as a separate file labeled 'Response to Reviewers'. This file does not need to include responses to any formatting updates and technical items listed in the 'Journal Requirements' section below.

* A marked-up copy of your manuscript that highlights changes made to the original version. You should upload this as a separate file labeled 'Revised Manuscript with Track Changes'.

* An unmarked version of your revised paper without tracked changes. You should upload this as a separate file labeled 'Manuscript'.

If you would like to make changes to your financial disclosure, competing interests statement, or data availability statement, please make these updates within the submission form at the time of resubmission. Guidelines for resubmitting your figure files are available below the reviewer comments at the end of this letter.

We look forward to receiving your revised manuscript.

Kind regards,

David J. Diemert, M.D.

Academic Editor

PLOS Neglected Tropical Diseases

Gabriel Rinaldi

Section Editor

PLOS Neglected Tropical Diseases

Shaden Kamhawi

co-Editor-in-Chief

PLOS Neglected Tropical Diseases

orcid.org/0000-0003-4304-636XX

Paul Brindley

co-Editor-in-Chief

PLOS Neglected Tropical Diseases

orcid.org/0000-0003-1765-0002

**Journal Requirements:**

If the reviewer comments include a recommendation to cite specific previously published works, please review and evaluate these publications to determine whether they are relevant and should be cited. There is no requirement to cite these works unless the editor has indicated otherwise.

1) Please ensure that the CRediT author contributions listed for every co-author are completed accurately and in full.

At this stage, the following Authors/Authors require contributions: Mable M. Mutengo, James Mwansa, Kelly Chisanga, Emmanuel Zulu, Namwiinga Rozaria Mulunda, James Muchinga, Esther Rodríguez, Sergio Sánchez, Lourdes Castro, Maria J. Perteguer, David Carmena, and Javier Sotillo. Please ensure that the full contributions of each author are acknowledged in the "Add/Edit/Remove Authors" section of our submission form.

The list of CRediT author contributions may be found here: https://journals.plos.org/plosntds/s/authorship#loc-author-contributions

2) We ask that a manuscript source file is provided at Revision. Please upload your manuscript file as a .doc, .docx, .rtf or .tex. If you are providing a .tex file, please upload it under the item type u2018LaTeX Source Fileu2019 and leave your .pdf version as the item type u2018Manuscriptu2019.

3) We do not publish any copyright or trademark symbols that usually accompany proprietary names, eg ©,  ®, or TM  (e.g. next to drug or reagent names). Therefore please remove all instances of trademark/copyright symbols throughout the text, including:

- ® on pages: 7, 10, and 14

- TM on page: 9.

4) Thank you for including an Ethics Statement for your study. Please include:

i) A statement that formal consent was obtained (must state whether verbal/written) OR the reason consent was not obtained (e.g. anonymity). NOTE: If child participants, the statement must declare that formal consent was obtained from the parent/guardian.].

5) Please amend your detailed Financial Disclosure statement. This is published with the article. It must therefore be completed in full sentences and contain the exact wording you wish to be published.

1) State what role the funders took in the study. If the funders had no role in your study, please state: "The funders had no role in study design, data collection and analysis, decision to publish, or preparation of the manuscript."

2) If any authors received a salary from any of your funders, please state which authors and which funders..

If you did not receive any funding for this study, please simply state: u201cThe authors received no specific funding for this work.u201d

**Reviewers' Comments:**

Reviewer's Responses to Questions

**Key Review Criteria Required for Acceptance?**

As you describe the new analyses required for acceptance, please consider the following:

**Methods**

-Are the objectives of the study clearly articulated with a clear testable hypothesis stated?

-Is the study design appropriate to address the stated objectives?

-Is the population clearly described and appropriate for the hypothesis being tested?

-Is the sample size sufficient to ensure adequate power to address the hypothesis being tested?

-Were correct statistical analysis used to support conclusions?

-Are there concerns about ethical or regulatory requirements being met?

Reviewer #1: The method used are appropriate and specific to the reported work.

Reviewer #2: See general notes

Reviewer #3: - The study area and population is clearly described. However, insufficient information about the sampling design is provided. A sample size calculation should be stated, with reasoning.

- Diagnostic procedures upon urine samples are generally well described, but I have the following suggestions: specify the Ct cut-off for positivity in the qPCR assay, confirm if an internal amplification control was used (if not, recognise that this may lead to false negative results).

- I have some concerns about the molecular characterisation of eggs. 64 individual eggs from 24 patients were analysed, and only 29 egg samples were subjected to ITS sequencing. Please clarify how the 24 patients and the 29 sequenced egg samples were selected. How many eggs were analysed per patient and why? How might this impact inferences? Given the restricted species scope of the T-ARMS-PCR assay, might some other species have been missed that are known to hybridise with S. haematobium in Zambia and neighbouring countries (e.g. S. mattheei). Please clarify was this assay chosen over assays that can detect more species in the S. haem group. Expansion on these topics is needed in the limitations section.

**********

**Results**

-Does the analysis presented match the analysis plan?

-Are the results clearly and completely presented?

-Are the figures (Tables, Images) of sufficient quality for clarity?

Reviewer #1: The analysis provided match the proposed analysis plan. The figures and images are appropriate to the output.

Reviewer #2: See general notes

Reviewer #3: - Demographics paragraph and Table 1: reporting mean age with CIs is a bit unconventional for descriptive demographics. Mean + SD or median with IQR would be more typical.

- Figure resolution is poor but this may be just an issue with the PDF sent to me for review.

- Figure 2 provides useful species-level context, but ITS-based phylogeny is limited. The tree presentation could be improved to include colour-based annotations to show location and species, which would make its interpretation easier for the reader.

**********

**Conclusions**

-Are the conclusions supported by the data presented?

-Are the limitations of analysis clearly described?

-Do the authors discuss how these data can be helpful to advance our understanding of the topic under study?

-Is public health relevance addressed?

Reviewer #1: The conclusion are supported by the data

Reviewer #2: See general notes

Reviewer #3: - Discussion lines 401-402 comment on diagnostic sensitivity and specificity but these are not presented in the results section.

- Molecular work shows that hybrids are present, but the limitations of the T-ARMS-PCR assay and ITS-based phylogeny prevent broader population-level conclusions.

- The limitations surrounding the molecular characterisation of eggs needs more discussion (see earlier comment in methods).

- The findings are clearly framed in the context of schistosomiasis control and elimination efforts.

**********

**Editorial and Data Presentation Modifications?**

Use this section for editorial suggestions as well as relatively minor modifications of existing data that would enhance clarity. If the only modifications needed are minor and/or editorial, you may wish to recommend “Minor Revision” or “Accept”.

Reviewer #1: (No Response)

Reviewer #2: See general notes

Reviewer #3: - There are some minor typographical errors: “Kfaue” should be corrected to “Kafue”, “sensitivity” instead of “sensibility”.

- Improved presentation of Figure 2 is recommended (as above).

**********

**Summary and General Comments**

Use this section to provide overall comments, discuss strengths/weaknesses of the study, novelty, significance, general execution and scholarship. You may also include additional comments for the author, including concerns about dual publication, research ethics, or publication ethics. If requesting major revision, please articulate the new experiments that are needed.

Reviewer #1: This study investigating on the prevalence and molecular identification of Schistosoma haematobium infection in peri-urban areas of Lusaka, Zambia, revealed very interesting patterns. The final observation provides the recommendation to consider One Health approach especially targeting zoonotic diseases. Animals and climate change are expected to have considerable contribution in the near future to intensified transmission in areas where control efforts through MDA would have been considered to have a success.

The authors refer to a higher rate of false positives as observed in the ELISA results, though referring to as residual antibodies being persist in serum/urine for several months after praziquantel treatment. This is a known phenomenon the reason ELISAs cannot be used for evaluation of treatment success. Further this provides a complication as post treatment the killed worms and eggs tend to provide as stimulation effect leading to elevated antibodies. The authors need to explain the need for such method use as I the design of the study.

What is the contribution of treatment and that effect of immunizing post MDA? In Kafue without the evidence of MDA there could be contribution of immune response development. Treatment is known to eliminate the infection, but the killed worm are also understood to contribute to evoking a specific immune response.

The clustering of Kafue and Chongwe sequences requires further explanation in addition to the indication of limited genetic differentiation across these sites. As the transmission may involve spreading across sites in each region and the generation of hybrids. Probably give an explanation to the high gene flow within these endemic areas.

Are the hybrids infectious? This could be a concern and would benefit from additional explanation.

The explanation on the hybridization between human and animal schistosome species can give pointers of possible hosts, creating zoonotic reservoirs that sustain transmission even when human treatment coverage is high. This calls for One Health and incorporation of zoonotic NTDs investigation in the control and prevention of schistosomiasis

Specific comments

Abstract: line 23 the word Sub-Saharan Africa should be written sub-Saharan Africa.

Line 250: on an ABI

Line 287: (111 from Chongwe and 97 from Kfaue) correct the spelling of Kafue?

Line 476: Firstly, the survey was restricted to paediatric populations in only two districts of Lusaka Province. The term paediatric should be used cautiously since the age group considered in this work is not paediatric.

Reviewer #2: The paper presents urogenital infection data and molecular data from eggs from humans. Although the infection data is informative the authors focus too much on the "hybrid" sorry with no robust data. The authors need to be very careful in their data analysis and interpretation related to this subject. The infection data is very useful and informative and I suggest the authors focus more on that. Currently the paper reads like it is lots of parts of studies brought together. All my comments are detailed below rather than be split by sections.

Abstract

Revise the abstract and summary according to the comments on the paper.

Introduction

Line 107: You need to explain the need for serological detection as it does not distinguish present and past infections so its use is limited.

Methods

Line 123: You need to explain what the further action was for those infected.

Line 104: “and later are infected” – they are infected at the time of water contact so revise.

Line 143: add the details of what the systematic random sampling method is.

Line 150: you do not explain how the microscopic leucocytes, biochemistry protein parameters were measured.

Line 156: were the eggs not stained?

Lines 159-180: this is a large area of work. Was this done in Zambia and add details as to why this method was used versus an available kit.

Line 210: In the results you do not mention what the Nanodrop informed you about your samples. Also, bear in mind the Nanodrop will measure any DNA and this includes human DNA so the quantification is not specific.

Line 223: What part of the urine were these taken from and how captured (it is not easy to find individual eggs and capture them. You say above that the urine was processed by microscopy and for molecular so was there urine left? Also, explain the procedure for the extraction or how is was adapted to eggs.

Lines 221-243: The T-ARMS description is fine but include all the information on the amplicon in a figure as it is not information in the main text.

Line 247: Why these bands? It is not clear how you would identify the SNP’s from these bands? Also, your NCBI accessions are all 518 bp long so how does that work with sequencing bands of only 487 and 234?

Line 253-255: You have to be very careful when BLASTing ITS sequences for Schisto species identification as the output is very often misinterpreted. It is better just to look at the species specific SNPs’.

Line 258-269: Running a phylogenetic analysis on ITS data for Sb, Sh and Sc is not informative due to the high level of conservation. It does not tell you anything more than what looking at the specific specific SNP’s tell you.

Line 256: See comments below on the sequences. The naming of the sequences on NCBI must be changed to reflect the incorrect assignment to Sh/Sc.

Line 261: When you are using ITS data from NCBI that is labelled as Sh/Sc or Sh/Sb you have to think about what that data is from and how informative it is. For example ITS data labelled Sh/Sb maybe from what is assumed to be a Sh/Sb hybrid that has a cox1 Sb and an ITS Sh so the ITS is just Sh and no different to a pure Sh. This is a common mistake many researcher make and just automatically assign data as from a hybrid because of the blast hit but if you look the BLAST hit will be 100% for the “hybrid” and for Sh. You must look at the data, think about it, think about what it is telling you and interpret it. All BLAST searches must be analysed informatively and not just be taken as whatever is at the top of the list.

Results

Line 299: why are leukocytes informative for your study?

Line 306-308, 319-320: do not repeat methods in the results

Line 326-330: it is not clear what the ELISA data adds? Also, there is a large section on this in the methods but a very small bit in the results?

Line 332: you mention correlation to age on 3.1 so you might want to change the structure.

Lines 332-356: It is really not clear what your data is saying as you combine all diagnostics and age and sex and region. Consider restricting this and think about how the data can be informative – are you comparing diagnostics or are you comparing infection levels (age, sex, region?).

Line 363: There is no text in your introduction to mention the “hybrid” topic. So many researcher now bring this into the studies without much thought of why or what it means. If you are going to present data on this topic you need to read the background literature, think about the meaning and make informed analysis of the data. You cannot make big statements without robust data.

Lines 361-381: There are major issues with this data and the way it has been analysed and interpreted. These are listed below.

1. You do not include how many eggs from each participant were analysed.

2. There is no cox1 data associated with these samples so you cannot make any robust inferences on species or “hybrid”. Be aware there is many papers now showing the the use of these basic molecular markers e.g. ITS are not robust for “hybrid” identification in terms of what a “hybrid” actually is and the biological consequences.

3. On your gel figure it is not clear what the samples represent and what did you use as your controls?

4. You phylogenetic analysis is not informative and see the notes above on using ITS data from NCBI and BLAST. You can see that there is no structuring for Sb, Sc. You do have some structuring for Sh and in fact that shows that if anything your samples are Sh.

5. Line 374 – 375 – that is a strange statement as why would you expect them to cluster? The authors should think about what they are trying to understand by the data analysis.

6. In this section you do not mention anything about the S. curassoni / Sh that you mention elsewhere in the paper and that you have put as an ID for some of your sequences. This is very strange.

7. All you sequences on Genbank are Sh ITS data. You must change the accessions that say they are Sh/Sc as they are not correct and will mislead other researcher.

8. The authors should think more about species and geographical locations. S. curassoni is only found in the West of Africa due to the specific snail it uses for transmission. It is not understood why the authors would identify data within their study as being S. curassoni without questioning it. they should also mention that the T-ARMS PCR has obviously given an in-correct profile for Sh.

9. Notes on your sequences:

• They are all Sh ITS

• Some of your sequences contain missing bp which is why your BLAST searches may have mislead you.

• They are longer than the fragments that you cut out of the gels.

It is not clear how the molecular ID relates to the study as it is mainly about diagnosis and prevalence levels. You do not need to just bring in molecular identifications as an add on especially if there is no hypothesis or robust methods.

Discussion

Lines 384-400: This is introduction and not discussion

Lines 407-416: This is why it is not clear why the ELISA was done. Also, these are not false positives. They are positive for antibodies due to past infection and it is not a phenomenon?

Line 446-447: This is a very bold statement with not robust data and you do not detail that in the results.

Line 449: The reference 12 also has mis identification of samples reported in it. There is no S. bovis in the data. They also only looked at ITS. Have a look at the data. As academics you should always look at the detail associated with unusual findings.

Line 450-455: Do you think your data is robust enough to state this?

Revise the rest of the discussion based on comments above.

Reviewer #3: The manuscript fills a knowledge gap surrounding urogenital schistosomiasis prevalence in peri-urban Zambia and identifies S. haematobium × S. curassoni hybrids. It will help to guide public health interventions such as MDA and will draw important attention to One Health control.

Further to the above comments, I have the following comments:

- Abstract: Under the results sub-heading, prevalence in each district should be stated.

- Abstract: Under the results sub-heading, the authors report on associations between S. haematobium infection and age + sex, but then clarify in the next sentence that many eggs were hybrids. Therefore, I suggest changing the original statement to say that urogenital schistosomiasis (not S. haematobium specifically) was associated with age.

- Introduction, lines 88-91 and 103-105 state historical prevalence estimates, including from Lusaka. Please clarify when estimates were from, as this will help set the scene for the current investigation.

- Some analysis of infection intensity and comparison between sites would be useful.

- An additional results table assessing diagnostic performance (sensitivity, specificity, PPV, NPV of microscopy and ELISA vs qPCR) would be welcome.

**********

PLOS authors have the option to publish the peer review history of their article (what does this mean?). If published, this will include your full peer review and any attached files.

If you choose “no”, your identity will remain anonymous but your review may still be made public.

**Do you want your identity to be public for this peer review?**  For information about this choice, including consent withdrawal, please see our Privacy Policy.

Reviewer #1: **Yes:**  Professor Takafira Mduluza

Reviewer #2: No

Reviewer #3: No

**Figure resubmission:**

While revising your submission, we strongly recommend that you use PLOS’s NAAS tool (https://ngplosjournals.pagemajik.ai/artanalysis) to test your figure files. NAAS can convert your figure files to the TIFF file type and meet basic requirements (such as print size, resolution), or provide you with a report on issues that do not meet our requirements and that NAAS cannot fix.

After uploading your figures to PLOS’s NAAS tool - https://ngplosjournals.pagemajik.ai/artanalysis, NAAS will process the files provided and display the results in the "Uploaded Files" section of the page as the processing is complete. If the uploaded figures meet our requirements (or NAAS is able to fix the files to meet our requirements), the figure will be marked as "fixed" above. If NAAS is unable to fix the files, a red "failed" label will appear above. When NAAS has confirmed that the figure files meet our requirements, please download the file via the download option, and include these NAAS processed figure files when submitting your revised manuscript.

**Reproducibility:**

To enhance the reproducibility of your results, we recommend that authors of applicable studies deposit laboratory protocols in protocols.io, where a protocol can be assigned its own identifier (DOI) such that it can be cited independently in the future. Additionally, PLOS ONE offers an option to publish peer-reviewed clinical study protocols. Read more information on sharing protocols at https://plos.org/protocols?utm_medium=editorial-email&utm_source=authorletters&utm_campaign=protocols

# Author comment: Author response to Decision Letter 1

**DOI:** 10.1371/journal.pntd.0014013.r002

10 Aug 2026

Attachment Submitted filename: Rebuttal_PNTD-D-26-00269.pdf

# Peer review: Decision Letter 1

**DOI:** 10.1371/journal.pntd.0014013.r003

**Authors:** David J. Diemert, Gabriel Rinaldi

24 Aug 2026

PNTD-D-26-00269R1Schistosoma haematobium infection among school age children in two peri-urban areas of Lusaka, Zambia: a cross-sectional studyPLOS Neglected Tropical DiseasesDear Dr. Sotillo,Thank you for submitting your manuscript to PLOS Neglected Tropical Diseases. After careful consideration, we feel that it has merit but does not fully meet PLOS Neglected Tropical Diseases's publication criteria as it currently stands. Therefore, we invite you to submit a revised version of the manuscript that addresses the points raised during the review process.Please submit your revised manuscript by Oct 23 2026 11:59PM. If you will need more time than this to complete your revisions, please reply to this message or contact the journal office at plosntds@plos.org. When you're ready to submit your revision, log on to https://www.editorialmanager.com/pntd/ and select the 'Submissions Needing Revision' folder to locate your manuscript file.Please include the following items when submitting your revised manuscript:* A letter that responds to each point raised by the editor and reviewer(s). You should upload this letter as a separate file labeled 'Response to Reviewers'. This file does not need to include responses to any formatting updates and technical items listed in the 'Journal Requirements' section below.* A marked-up copy of your manuscript that highlights changes made to the original version. You should upload this as a separate file labeled 'Revised Manuscript with Track Changes'.* An unmarked version of your revised paper without tracked changes. You should upload this as a separate file labeled 'Manuscript'. If you would like to make changes to your financial disclosure, competing interests statement, or data availability statement, please make these updates within the submission form at the time of resubmission. Guidelines for resubmitting your figure files are available below the reviewer comments at the end of this letter.As the corresponding author, your ORCID iD is verified in the submission system and will appear in the published article. PLOS supports the use of ORCID, and we encourage all coauthors to register for an ORCID iD and use it as well. Please encourage your coauthors to verify their ORCID iD within the submission system before final acceptance, as unverified ORCID iDs will not appear in the published article. Only the individual author can complete the verification step; PLOS staff cannot verify ORCID iDs on behalf of authors.

We look forward to receiving your revised manuscript.

Kind regards,David J. Diemert, M.D.Academic EditorPLOS Neglected Tropical DiseasesGabriel RinaldiSection EditorPLOS Neglected Tropical Diseases

Shaden Kamhawi

co-Editor-in-Chief

PLOS Neglected Tropical Diseases

orcid.org/0000-0003-4304-636XX

Paul Brindley

co-Editor-in-Chief

PLOS Neglected Tropical Diseases

orcid.org/0000-0003-1765-0002

**Reviewers' comments:**

Reviewer's Responses to Questions

**Key Review Criteria Required for Acceptance?**

As you describe the new analyses required for acceptance, please consider the following:

**Methods**

-Are the objectives of the study clearly articulated with a clear testable hypothesis stated?

-Is the study design appropriate to address the stated objectives?

-Is the population clearly described and appropriate for the hypothesis being tested?

-Is the sample size sufficient to ensure adequate power to address the hypothesis being tested?

-Were correct statistical analysis used to support conclusions?

-Are there concerns about ethical or regulatory requirements being met?

Reviewer #1: Clear and detailed methods and presentation

Reviewer #2: See general comments

Reviewer #3: (No Response)

**********

**Results**

-Does the analysis presented match the analysis plan?

-Are the results clearly and completely presented?

-Are the figures (Tables, Images) of sufficient quality for clarity?

Reviewer #1: The results are presented in a proper sequence and clearly.

Reviewer #2: See general comments

Reviewer #3: (No Response)

**********

**Conclusions**

-Are the conclusions supported by the data presented?

-Are the limitations of analysis clearly described?

-Do the authors discuss how these data can be helpful to advance our understanding of the topic under study?

-Is public health relevance addressed?

Reviewer #1: The conclusion is in line with the set objectives and the general objective and findings.

Reviewer #2: See general comments

Reviewer #3: (No Response)

**********

**Editorial and Data Presentation Modifications?**

Use this section for editorial suggestions as well as relatively minor modifications of existing data that would enhance clarity. If the only modifications needed are minor and/or editorial, you may wish to recommend “Minor Revision” or “Accept”.

Reviewer #1: (No Response)

Reviewer #2: (No Response)

Reviewer #3: (No Response)

**********

**Summary and General Comments**

Use this section to provide overall comments, discuss strengths/weaknesses of the study, novelty, significance, general execution and scholarship. You may also include additional comments for the author, including concerns about dual publication, research ethics, or publication ethics. If requesting major revision, please articulate the new experiments that are needed.

Reviewer #1: Study area and population

Please provide the GPS locations of the study areas.

The statement requires elaboration as to the reason ELISA results are as indicator of cumulative population-level exposure to Schistosoma spp. rather than a direct measure of current infection status. How is this explained where ELISA is believed for distinguishing past from current exposure? What does cumulative mean?

A clearer explanation should be made in the discussion on the hierarch of the diagnostic methods: microscopy, ELISA, and qPCR - high analytical sensitivity for detecting parasite DNA, qPCR showed the highest sensitivity (Table 4).

Reviewer #2: The authors have greatly improved the paper, and the omission of the genetic data allows the findings to be better presented. It now reads very nicely. There are additional comments to address and data to include. The data are informative and important for Schistosomiasis control in Zambia. Comments are detailed below.

Line 146-155 – It is not very clear how the randomisation and random sampling were done. Maybe some references could support the statements. Also, line 155/6 – what do you mean by sampling interval?

Line 172 – The production of the ELISA reagents should be mentioned in the introduction, and more explanation of why this was done should be included, e.g. that previous studies have shown that it is better than other methods such as SEA. This part should also come under a general heading of ELISA. It is not clear why this method has not be accepted more widely. For example, ELISA is used as a diagnostic for traveller's infections, etc., and SEA is used.

Line 230 – the inclusion of an internal control has been added, but no optimisation is stated, and no references are given. Typically, very low levels of human DNA are detected in urine. The authors should show some amplification curves for the assay. Additionally, no standard curve is used for the qPCR, and this should be discussed in the paper. It allows quality control of the qPCR and evaluation of what the very high CT values represent, especially as CT values above 35 should be evaluated carefully.

Line 256 – should ELISA be included in the composite reference, as it cannot distinguish between active and past infection and therefore cannot be compared to the other tests?

Some intensity data should be brought into the results based on CT values and egg counts. This would be very informative in comparison to morbidity assessments, e.g. haematuria, etc. If this data is not available, then you should state this in the methods, and that the data were treated as binary, e.g. positive and negative. CT values are needed to assess the assay’s potential specificity.

Line 351 – the authors should discuss what trace amounts of DNA would mean in terms of an infection, as it would not be eggs?

The ELISA data needs some thought: although it detects past infections, it should also detect current infections, which is what qPCR also detects. You could also discuss the possible false positives for qPCR at very high CT. Most studies implement a CT cutoff of at least 35 due to false positives.

Line 430 – if you are going to mention hybrids, you should define what you mean by hybrids and also look at the balanced literature that shows most hybrids in West Africa are ancient forms and do not constitute a zoonotic risk. For Zambia, you should talk about the S. mattheei issue rather than S. bovis and S. curassoni, which are not present there. You should state that there are many unknowns about the zoonotic risk. Very few animals, if any, have been found to be infected with S. haematobium and/or hybrids.

Overall, the discussion could be reduced to make it more concise and focused on the data presented.

Reviewer #3: The authors have addressed everything highlighted in my previous review. This is an interesting article with important implications for the surveillance and control of urogenital schistosomiasis. I have no further concerns.

**********

PLOS authors have the option to publish the peer review history of their article (what does this mean?). If published, this will include your full peer review and any attached files.

If you choose “no”, your identity will remain anonymous but your review may still be made public.

**Do you want your identity to be public for this peer review?**  For information about this choice, including consent withdrawal, please see our Privacy Policy.

Reviewer #1: **Yes:**  Professor Takafira Mduluza

Reviewer #2: No

Reviewer #3: No

[NOTE: If reviewer comments were submitted as an attachment file, they will be attached to this email and accessible via the submission site. Please log into your account, locate the manuscript record, and check for the action link "View Attachments". If this link does not appear, there are no attachment files.] **Figure resubmission:**   While revising your submission, we strongly recommend that you use PLOS’s NAAS tool (https://ngplosjournals.pagemajik.ai/artanalysis) to test your figure files. NAAS can convert your figure files to the TIFF file type and meet basic requirements (such as print size, resolution), or provide you with a report on issues that do not meet our requirements and that NAAS cannot fix.

After uploading your figures to PLOS’s NAAS tool - https://ngplosjournals.pagemajik.ai/artanalysis, NAAS will process the files provided and display the results in the "Uploaded Files" section of the page as the processing is complete. If the uploaded figures meet our requirements (or NAAS is able to fix the files to meet our requirements), the figure will be marked as "fixed" above. If NAAS is unable to fix the files, a red "failed" label will appear above. When NAAS has confirmed that the figure files meet our requirements, please download the file via the download option, and include these NAAS processed figure files when submitting your revised manuscript. **Reproducibility:**  To enhance the reproducibility of your results, we recommend that authors of applicable studies deposit laboratory protocols in protocols.io, where a protocol can be assigned its own identifier (DOI) such that it can be cited independently in the future. Additionally, PLOS ONE offers an option to publish peer-reviewed clinical study protocols. Read more information on sharing protocols at https://plos.org/protocols?utm_medium=editorial-email&utm_source=authorletters&utm_campaign=protocols

# Author comment: Author response to Decision Letter 2

**DOI:** 10.1371/journal.pntd.0014013.r004

26 Aug 2026

Attachment Submitted filename: Rebuttal_PNTD-D-26-00269.docx

# Editor report: Decision Letter 2

**DOI:** 10.1371/journal.pntd.0014013.r005

**Authors:** David J. Diemert, Gabriel Rinaldi

21 Sep 2026

Dear Dr. Sotillo,

We are pleased to inform you that your manuscript 'Schistosoma haematobium infection among school age children in two peri-urban areas of Lusaka, Zambia: a cross-sectional study' has been provisionally accepted for publication in PLOS Neglected Tropical Diseases.

Before your manuscript can be formally accepted you will need to complete some formatting changes, which you will receive in a follow up email. A member of our team will be in touch with a set of requests.

Please note that your manuscript will not be scheduled for publication until you have made the required changes, so a swift response is appreciated.

IMPORTANT: The editorial review process is now complete. PLOS will only permit corrections to spelling, formatting or significant scientific errors from this point onwards. Requests for major changes, or any which affect the scientific understanding of your work, will cause delays to the publication date of your manuscript.

Should you, your institution's press office or the journal office choose to press release your paper, you will automatically be opted out of early publication. We ask that you notify us now if you or your institution is planning to press release the article. All press must be co-ordinated with PLOS.

Thank you again for supporting Open Access publishing; we are looking forward to publishing your work in PLOS Neglected Tropical Diseases.

Best regards,

David J. Diemert, M.D.

Academic Editor

PLOS Neglected Tropical Diseases

Gabriel Rinaldi

Section Editor

PLOS Neglected Tropical Diseases

Shaden Kamhawi

co-Editor-in-Chief

PLOS Neglected Tropical Diseases

orcid.org/0000-0003-4304-636XX

Paul Brindley

co-Editor-in-Chief

PLOS Neglected Tropical Diseases

orcid.org/0000-0003-1765-0002

***********************************************************

# Editor report: Acceptance letter

**DOI:** 10.1371/journal.pntd.0014013.r006

**Authors:** David J. Diemert, Gabriel Rinaldi

Dear Dr. Sotillo,

We are delighted to inform you that your manuscript, "Schistosoma haematobium infection among school age children in two peri-urban areas of Lusaka, Zambia: a cross-sectional study," has been formally accepted for publication in PLOS Neglected Tropical Diseases.

We have now passed your article onto the PLOS Production Department who will complete the rest of the publication process. All authors will receive a confirmation email upon publication.

The corresponding author will soon be receiving a typeset proof for review, to ensure errors have not been introduced during production. Please review the PDF proof of your manuscript carefully, as this is the last chance to correct any scientific or type-setting errors. Please note that major changes, or those which affect the scientific understanding of the work, will likely cause delays to the publication date of your manuscript. Note: Proofs for Front Matter articles (Editorial, Viewpoint, Symposium, Review, etc...) are generated on a different schedule and may not be made available as quickly.

Soon after your final files are uploaded, the early version of your manuscript will be published online unless you opted out of this process. The date of the early version will be your article's publication date. The final article will be published to the same URL, and all versions of the paper will be accessible to readers.

For Research Articles, you will receive an invoice from PLOS for your publication fee after your manuscript has reached the completed accept phase. If you receive an email requesting payment before acceptance or for any other service, this may be a phishing scheme. Learn how to identify phishing emails and protect your accounts at https://explore.plos.org/phishing.

Thank you again for supporting open-access publishing; we are looking forward to publishing your work in PLOS Neglected Tropical Diseases.

Best regards,

Shaden Kamhawi

co-Editor-in-Chief

PLOS Neglected Tropical Diseases

Paul Brindley

co-Editor-in-Chief

PLOS Neglected Tropical Diseases
